# 4-Isobutylmethcathinone—A Novel Synthetic Cathinone with High In Vitro Cytotoxicity and Strong Receptor Binding Preference of Enantiomers

**DOI:** 10.3390/ph15121495

**Published:** 2022-11-30

**Authors:** Martin Paškan, Silvie Rimpelová, Vladimíra Svobodová Pavlíčková, Dita Spálovská, Vladimír Setnička, Martin Kuchař, Michal Kohout

**Affiliations:** 1 Department of Organic Chemistry, University of Chemistry and Technology Prague, 166 28 Prague, Czech Republic; 2 Department of Biochemistry and Microbiology, University of Chemistry and Technology Prague, 166 28 Prague, Czech Republic; 3 Department of Analytical Chemistry, University of Chemistry and Technology Prague, 166 28 Prague, Czech Republic; 4 Forensic Laboratory of Biologically Active Substances, Department of Chemistry of Natural Compounds, University of Chemistry and Technology, 166 28 Prague, Czech Republic

**Keywords:** synthetic cathinones, cytotoxicity, chiral drugs, chiral separation, neurotoxicity, enantiomer-selective binding to receptors, receptor binding studies, density functional theory calculations, absolute configuration

## Abstract

New psychoactive substances and among them synthetic cathinones represent a significant threat to human health globally. However, within such a large pool of substances derived from a natural compound ((*S*)-cathinone), substances with important pharmaceutical uses can be identified, as already documented by bupropione. Therefore, this work aimed to find a synthetic pathway for a novel synthetic cathinone, namely 4-isobutylmethcathinone, and describe its spectroscopic properties and biological activity in vitro. Since cathinones comprise a chiral center in their structure, a method for chiral separation of the substance was elaborated using high-performance liquid chromatography on an analytical and preparative scale. Preparative enantioseparation on a polysaccharide column provided a sufficient amount of the drug for the chiroptical studies leading to the determination of the absolute configuration of enantiomers as well as for their subsequent in vitro cytotoxicity study. The cytotoxicity induced by 4-isobutylmethcathinone was determined in human cells derived from the urinary bladder (5637), neuroblastoma (SH-SY5Y), microglia (HMC-3), and hepatocellular carcinoma (Hep G2), in which the IC_50_ values after 72 h reached an 18–65 µM concentration. This is significantly higher cytotoxicity in comparison with other synthetic cathinones. In the receptor binding studies, a significant difference in the agonistic effect on dopamine and adrenergic receptors of individual enantiomers was observed. The lack of binding affinity towards the serotonin receptors then relates 4-isobutylmethcathinone to the family of monoamine drugs, such as 3,4-methylenedioxymathamphetamine (ecstasy, MDMA).

## 1. Introduction

**Figure 1:**
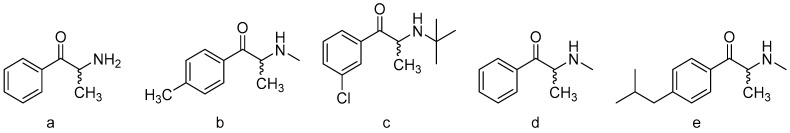
Chemical structures of (**a**) cathinone, (**b**) mephedrone, (**c**) bupropion, (**d**) methcathinone, and (**e**) 4-isobutylmethcathinone (IBMCat).

Synthetic cathinones are phenylalkylamine derivates possessing a carbonyl group at the β-position to the amino group in the vicinity of the aromatic unit (Figure 1a) [1,2]. The drug scaffold originates from the natural alkaloid *S*-cathinone present in the leaves of the plant *Catha edulis*. It was reported that this alkaloid possessed an amphetamine-like effect [3]. More specifically, cathinone is a mixed-acting sympathomimetic central nervous system stimulant [4]. However, it is not stable and relatively readily decomposes to less active cathine [5]. Therefore, clandestine laboratories work on structural modification of the natural scaffold to improve its stability. Hand in hand with the structural modification also comes the change in psychostimulative properties of the new synthetic cathinone [6]. Indeed, it has been documented that structural variation of synthetic cathinones leads to a difference in their pharmacokinetics and the way they affect the function of monoamine transporters [7,8]. Particular attention has been paid to neuropharmacology of mephedrone in comparison to amphetamines [9], mephedrone metabolites [10,11], and variation in the effects of its enantiomers [12,13].

Mephedrone (Figure 1b) is still one of the most popular and prevalent cathinones, which is available on the market, although it was placed among the controlled substances in 2010. Similar to other cathinones and amphetamines, mephedrone interacts mainly with the dopamine, norepinephrine, and serotonin transporters (DAT, NET, and SERT, respectively) [8,10,11]. These membrane proteins are involved in the reuptake of the corresponding neurotransmitters, thereby regulating their diffusion and, consequently, binding to the receptors [14,15]. Therefore, DAT, NET, and SERT possess an important role in brain signaling and represent a target for several pharmacological agents, either in medical use (antidepressants), or recreationally misused (psychostimulants) [15,16]. Synthetic cathinones belong more to the psychostimulants; however, bupropion (Figure 1c) is known as an antidepressant with the main use these days as an aid in smoking cessation. Another substance, 4-methcathinone (Figure 1d), was in use as an antidepressant in the Soviet Union from 1930; however, it was placed among the controlled substances in the 1990s due to its widespread abuse [5]. More recently, it was documented that 4-methcathinone possesses a similar neurochemical profile to mephedrone. However, in comparison to mephedrone, it possesses a weaker effect on extracellular dopamine, therefore, it is assumed that it displays a more serotonergic profile of activity [10]. Therefore, the chemical structure of synthetic cathinones seems to play an important role in their action.

Given the synthetic route, synthetic cathinones are typically prepared as racemates, i.e., both possible enantiomers originating from a center of chirality in their molecular structure are present. It has already been thoroughly reviewed that enantiomers possess different effects in the chiral environment of a living organism [17,18,19]. Similar observation has been made for synthetic cathinones. In the case of mephedrone, a well-known representative of synthetic cathinones, the effect of *R*- and *S*-enantiomers on DAT and NET was the same, both behaved as releasers with similar potency. On the other hand, *S*-mephedrone is a SERT releaser with 40 times higher potency than *R*-mephedrone.

Apart from the modulation of neural network function, synthetic cathinones also exhibit a toxic effect; the structure–cytotoxicity relationship was studied in vitro in a model cell line from neuroblastoma (SH-SY5Y cells) using 13 different cathinones [20]. It was documented that cathinone cytotoxicity increased with the increasing number of methyl substituents on the central aromatic ring as well as with the increasing length of an aliphatic chain connected to the amino group. On the other hand, upon increasing the size or the number of alkyl substituents in the amino group, reduced in vitro cytotoxicity towards the model SH-SY5Y cells was observed [20]. A similar effect was documented also for human cells derived from hepatocellular carcinoma (Hep G2) [21]. Not only the number and position of the substituents but also their orientation in space can be of importance leading to enantiomer-specific toxicity of a drug [22]. Several examples of such behavior have already been reported, among others, higher toxicity of (*S*)-(+)-pentedrone and (*R*)-(+)-methylone to SH-SY5Y cells in comparison to their enantiomers [23], and enantiomer-specific cytotoxicity of (*R*)-(−)-pentedrone towards human stem-cell-derived hepatocyte-like cells [24]. This difference in the action of pentedrone enantiomers can be explained by different mechanisms inducing cell toxicity: oxidative stress and toxic metabolite production in cells of neuronal and hepatic origin, respectively.

Since both the chemical structure and chirality of cathinones seem to play a pivotal role in their biological function, this study aimed to prepare 4-isobutylmethcathinone (IBMCat, Figure 1e) in the enantiomerically pure form. This novel substance possesses an isobutyl fragment, which is known for its application in the non-steroidal anti-inflammatory drug ibuprofen, in combination with the cathinone pharmacophore. The isobutyl fragment could enhance blood–brain barrier penetration thereby increasing the biological effect of the drug [10].

## 2. Results and Discussion

### 2.1. Synthesis and Characterization

**Figure 2:**
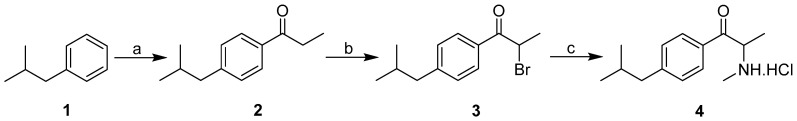
General synthesis of 4-isobutylmethcathinone (**4**), reaction conditions: (**a**) propionyl chloride, AlCl_3_, CH_2_Cl_2_, 0 °C, 1 h; (**b**) CuBr_2_, EtOAc, reflux, 4 h; (**c**) NH_2_CH_3_, THF, room temperature, 4 h, then aq. HCl.

A detailed synthesis of 4-isobutylmethcathinone hydrochloride (**4**) is presented in the ESI. Generally, the synthesis started from isobutyl benzene **1**, which was acylated using Friedel-Crafts reaction to afford ketone **2** that was then selectively brominated to α-position to form α-bromo derivative **3** (Figure 2). This derivative was transformed by substitution with methylamine to the target compound **4**.

### 2.2. Chiral Separation

**Figure 3:**
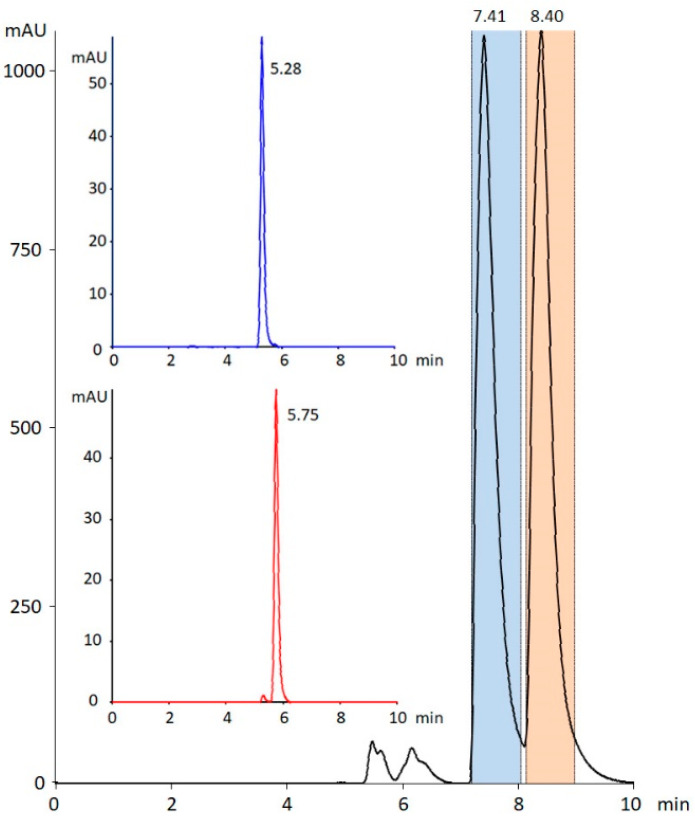
Preparative enantioseparation of 4-IBMCat with the marked boundaries for the collection of the first and the second eluting enantiomers. The insets show the analytical control of the optical purity of the collected enantiomers: 4-IBMCat 1 (**blue**, **top**) and 4-IBMCat 2 (**red**, **bottom**).

To determine the absolute configuration of the enantiomers and, subsequently, study their biological activity, the chiral resolution of the synthesized racemic mixture was developed de novo. The selection of chiral stationary phases and the optimization of the mobile phase composition are summarized in ESI. Although the screening was performed with polysaccharide columns from YMC and experimental strong chiral cation-exchange columns developed in our laboratory, based on our previous results in the chiral separation of cathinone derivatives [25,26,27,28,29], we decided to employ the polysaccharide chiral stationary phase Chiralpak IA. It should be noted that the chiral selector utilized in both types of polysaccharide columns is the same (tris{3,5-dimethylphenylcarbamoyl}amylose). The developed preparative method enabled chiral resolution of more than 30 mg of each enantiomer of IBMCat with the purity and enantiomeric excess (ee) of >99% (>98% ee) and >97.5% (>95% ee) for the first and second eluting enantiomer, respectively (Figure 3).

### 2.3. Assignment of the Absolute Configuration

**Figure 4:**
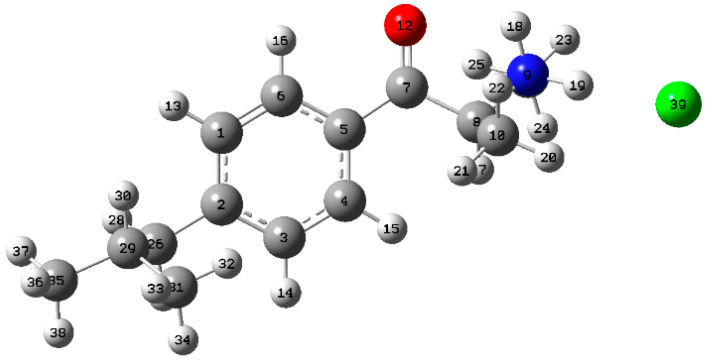
The structure of the most stable conformer of (*S*)-4-isobutylmethcathinone hydrochloride in MeOH simulated at B3LYP/6-311++G(d,p)/CPCM level.

After a conformational search via DFT calculations, five stable conformers were found (Table 1). The relative abundance of the most stable conformer (Figure 4) calculated using the Boltzmann distribution was 38% and its structure was stabilized by a hydrogen bond between the oxygen of the carbonyl group and hydrogen of the amine group. For the rest of the stable conformer structures, see ESI (Figure S9).

**Figure 5:**
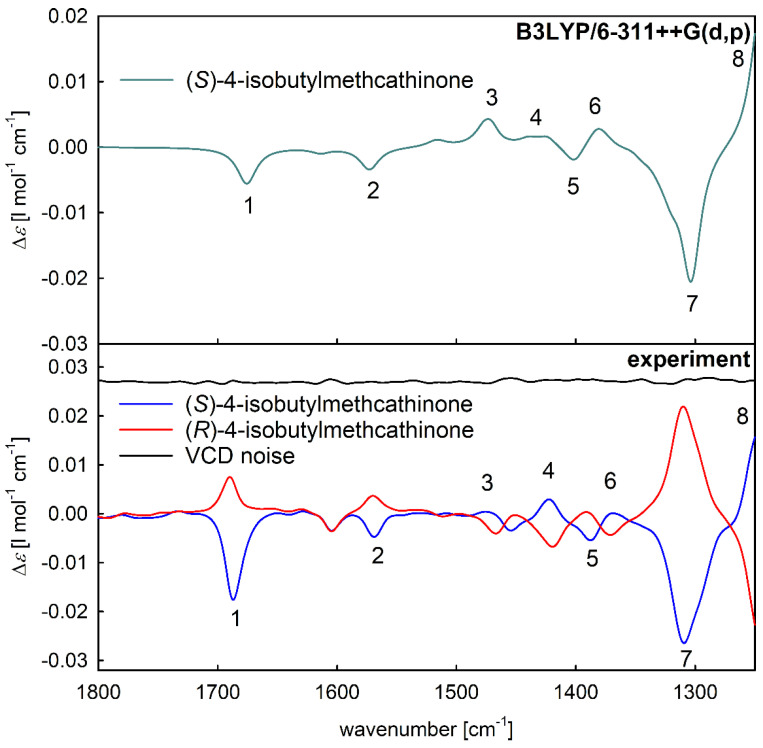
Comparison of the VCD spectra of 4-isobutylcathinone in MeOD-*d*_4_: the simulated spectrum of (*S*)-enantiomer calculated at the B3LYP/6-311++G(d,p) level (**top**) and experimental spectra with the spectrum of noise which was offset for clarity (**bottom**).

The mirror-image experimental VCD spectra (Figure 5 bottom, blue and red spectra) confirmed that the two separated molecules were individual enantiomers of 4-isobutylcathinone. After a comparison of the spectral pattern of experimental spectra with the simulated VCD spectrum of (*S*)-enantiomer (Figure 5, top), the absolute configuration of the enantioseparated samples was assigned as (*S*)-4-isobutylcathinone for the blue spectrum (Figure 5, bottom) and (*R*)-enantiomer for the red spectrum due to the relative magnitudes and signs of the corresponding VCD bands. To see the corresponding experimental and simulated IR absorption spectra, see ESI (Figure S10).

**Figure 6:**
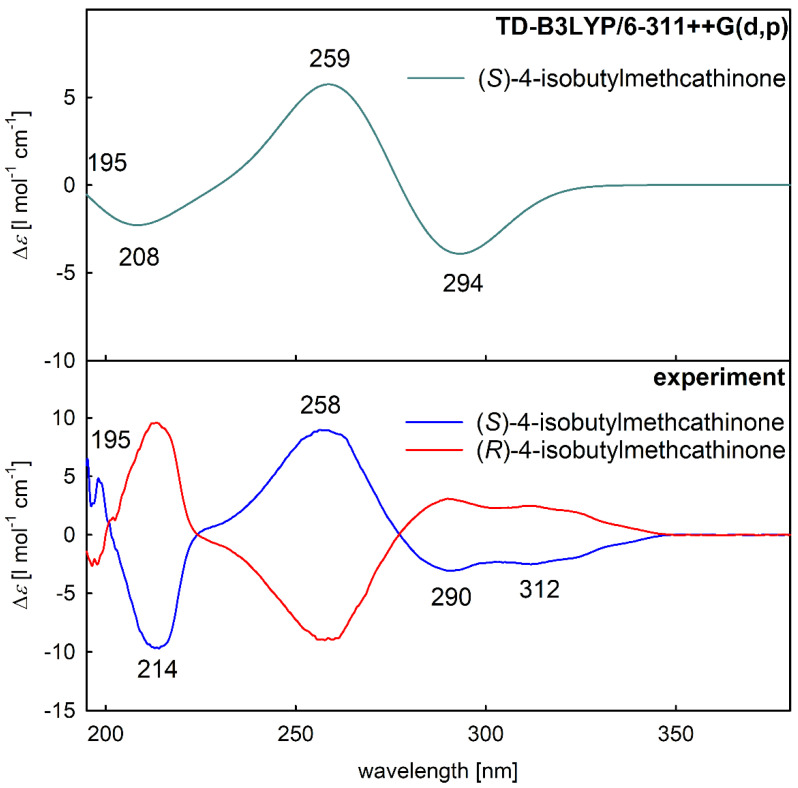
Comparison of the ECD spectra of 4-isobutylcathinone in MeOH: the simulated spectrum of (*S*)-enantiomer at the TD-B3LYP/6-311++G(d,p) level (**top**) and experimental spectra (**bottom**).

Similar to VCD analysis, the opposite sign of the experimental ECD spectra (Figure 6, bottom) confirmed the presence of two individual enantiomers. The mutual spectral pattern of the blue experimental spectrum (Figure 6, bottom) and the simulated spectrum of (*S*)-enantiomer (Figure 6, top) verified the assignment of the absolute configuration also by ECD spectroscopy. Thus, the first eluting enantiomer IBMCat1 corresponds to *S*-enantiomer and the second eluting enantiomer IBMCat2 corresponds to *R*-enantiomer on the utilized polysaccharide-based chiral stationary phase. To see the corresponding experimental and simulated UV absorption spectra, see ESI (Figure S11).

### 2.4. Cytotoxicity of 4-Isobutylmethcathinone and Its Enantiomers

Based on the novelty of the IMBcat compound, we aimed to evaluate its in vitro cytotoxicity in selected model cell lines as well as to determine the toxicity of the IBMcat enantiomers, since both the chemical structure and chirality of compounds could play a role in their bioactivity (Table 2). The cytotoxic profile of the IBMcat was determined by a WST-1 viability assay after 72-h treatment. For this purpose, cell lines derived from distinct tissues, often affected by NPSs, were chosen, i.e., cells of kidney, hepatic, neural, urinary, and intestinal origin as well as keratinocytes and primary fibroblasts (HEK 293T, Hep G2, HMC-3, SH-SY5Y, 5637, CaCo-2, HaCaT, MRC-5, and BJ, respectively). The cytotoxicity of IBMcat and its enantiomers was concentration- and cell-line dependent, the IC_50_ values, which were in the micromolar range, are given in Table 2. Surprisingly, big differences in the cytotoxicities in different cell lines were detected. The highest cytotoxicity of IBMcat was detected in human cells derived from urinary bladder carcinoma (5637), neuroblastoma (SH-SY5Y), microglia (HMC-3, immortalized), hepatocarcinoma (Hep G2), and mouse fibroblasts (L929; immortalized), in which the IC_50_ values reached from 18 to 65 µM concentration. This was followed by embryonic kidney cells HEK 293T (immortalized), in which the toxicity ranged between a concentration of 123 and 192 µM. For the rest of the cell lines, i.e., keratinocytes HaCaT (immortalized), colon carcinoma cells CaCo-2, and noncancerous fibroblasts MRC-5 and BJ, the toxicity was markedly lower with the IC_50_ ranging from 329 to 749 µM.

The results indicate that for the studied cell lines, there was no considerable effect of chirality on the cytotoxicity of the drug, as both enantiomers showed similar IC_50_ values. However, it is important to note the difference observed for HEK 293T cells, where the racemic mixture exhibited substantially higher toxicity than the individual enantiomers. It is assumed that such behavior could be caused by a difference in the metabolism of the enantiomers and the concerted toxic effects of the metabolites on the kidney cells.

Generally, the IC_50_ values below 100 μM can be considered rather unusual for cathinones, and therefore, the toxicity of IBMCat is relatively high. This is because reported values for, e.g., mitochondrial dysfunction in neuronal differentiated SH-SY5Y cells after 24-h exposure for alpha-PVP, which is considered to be highly toxic cathinone [20], have been found above 100 mM, as determined by ToxiLight BioAssay [30]. Similarly, for 3,4-DMMC, which has also been reported as highly toxic [20], the IC_50_ value exceeds 1 mM after 24 h of exposure for SH-SY5Y cells. Although our experiments were based on 72-h exposure and the IC_50_ values reported herein could be, therefore, substantially lower than those reported in the literature, the toxicity of IBMCat can still be considered exceptional, *vide infra*.

### 2.5. Fluorescence Microscopy

**Figure 7:**
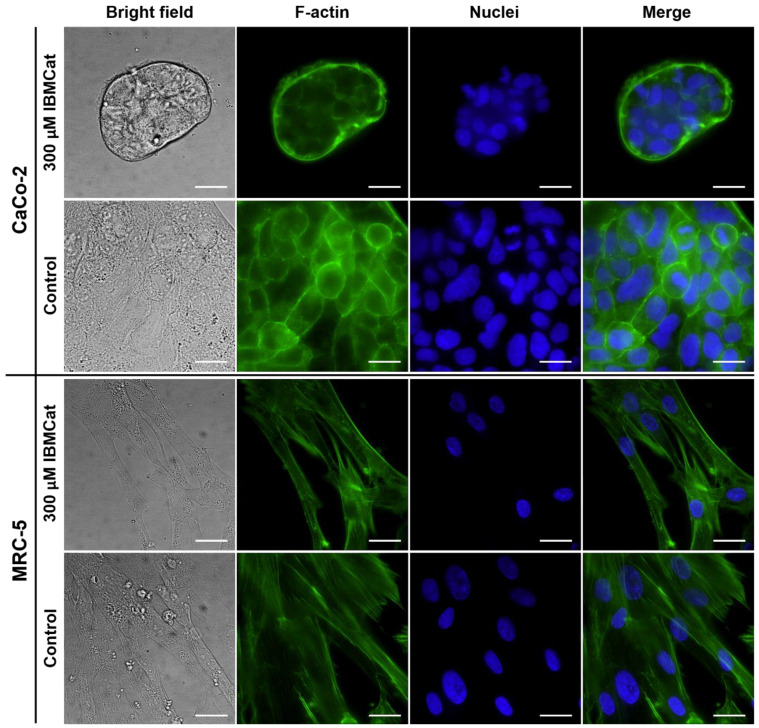
Fluorescence microscopy images of human cells derived from colon carcinoma (CaCo-2) and human primary fibroblasts (MRC-5) treated with 100–500 µM concentration of 4-isobutylmethcathinone (IBMCat) for 24 h. Individual columns from left: bright-field images, F-actin labeled with phalloidin-Atto 488, cell nuclei stained with DAPI, and merges of the fluorescent images. The scale bars correspond to 20 µm.

In addition to the evaluation of IBMcat cytotoxicity in various cell lines, its impact on cell morphology was also examined in CaCo-2 and MRC-5 cells after 24-h treatment. F-actin and cell nuclei were visualized in these cells treated with 100, 300, and 500-µM concentrations of IBMcat and its enantiomers. IBMcat (Figure 7) induced a considerable change in CaCo-2 cell morphology already from 300-µM concentration, the cells were smaller, bore roundish shapes, and grew in clusters when compared to control untreated cells. This could be ascribed to the substantial toxicity of IBMcat manifested already after 24 h at this concentration. No difference was observed between the individual enantiomers. Regarding MRC-5 cells, IBMcat did not induce any shape-related effect in the cells, as it is apparent from Figure 7, in which there are cells of prolonged shape typical for fibroblasts plus they did not differ from the control. However, in the IBMcat-treated samples, there were considerably lesser cells than in the control, which is in agreement with the cytotoxicity data.

### 2.6. Receptor Binding Studies

Cathinones and their derivatives, like amphetamines, are generally considered to stimulate the central nervous system (sympathomimetics). The mechanism of their action is based on structural similarity to the natural monoamines noradrenaline (NE), dopamine (DA), and serotonin (SR). It has been reported that cathinones act primarily as inhibitors of the monoamine reuptake due to the inhibition of relevant transporters such as NET, DAT, and SERT. In some studies, the role of cathinones has been also described as inverse agonists, which, when bound to the transporter, alter its conformation and, thus, reverse the flow of monoamines, which are subsequently effluxed outside a cell [31,32,33]. Cathinones can also act as substrates that are transported inside cells, in which then, upon their action, monoamines are effluxed out of the storage vesicles, which is followed by passive transport of monoamines out of the cells. Further action of cathinones may be mediated by their interaction with G-protein coupled receptors (GPCRs) including dopamine receptor (DAR), serotonin receptor (SRR), adrenergic receptor (ADR), trace amine-associated receptor 1 (TAAR-1), and others [30,34,35,36]. For this reason, we hypothesized that monoamine receptors or transporters may be the preferred site of action of the newly synthesized cathinones.

**Figure 8:**
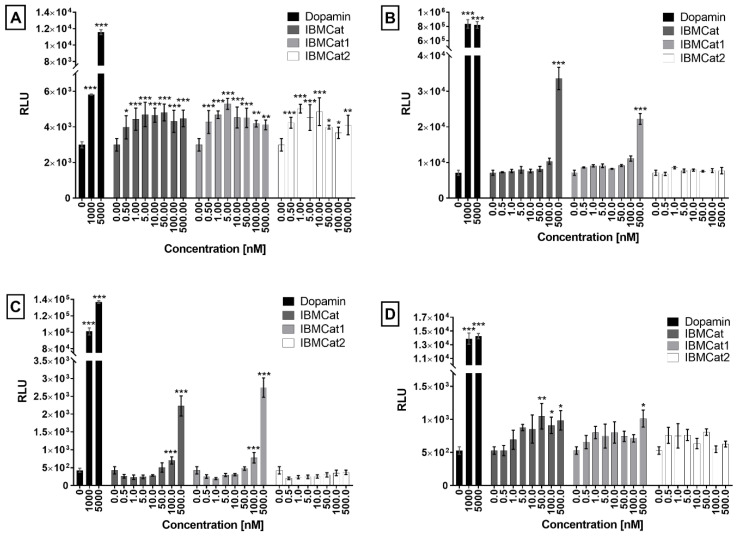
β-Arrestin 2 recruitment PRESTO-Tango assay: activation of the dopamine receptors. The plots represent the luminescence level of oxyluciferin (relative light unit, RLU) arisen in HTLA cells transfected with pDNA encoding the dopamine receptors and treated with 4-isobutylmethcathinone (IBMCat) and its enantiomers IBMCat1 an IBMCat2 at 0–500 nM concentration. As a control, a ligand of dopamine receptors (DAR), dopamine (1 and 5 µM concentration), was used. (**A**) DAR1, (**B**) DAR2, (**C**) DAR3, and (**D**) DAR4. The error bar represents the standard deviation from four replicates. The data were processed and evaluated by GraphPad Prism Software using one-way ANOVA: * *p* < 0.05; ** *p* < 0.01; *** *p* < 0.001.

However, binding to a specific receptor/transporter and, thus, the mechanism of action of a given cathinone always depends on the specific structure of the evaluated compound [37]. In this study, the binding activity of IBMcat and its enantiomers was verified by PRESTO-Tango (parallel receptor expression and screening via transcriptional output, with transcriptional activation following arrestin-2 translocation) assay using the HTLA cell line stably producing β-arrestin-2 in a fusion with TEV and luciferase production driven by tTA. For this purpose, the HTLA cells were transiently transfected with plasmid DNA encoding the dopamine receptors type 1-4 (DAR1-4), which also contain the TEV protease cleavage site followed by tTA at the 3’-end (DAR1-Tango, DAR2-Tango, DAR3-Tango, DAR4-Tango) [38]. In the case of the receptor activation by a ligand, in transfected cells, the receptor is desensitized by β-arrestin fused to TEV protease. This brings the TEV protease close to the site that specifically cleaves and, thus, the transcription factor can be cleaved. The transcription factor then translocates to the cell nucleus, in which it triggers the transcription of the target gene for luciferase production which can be determined by the light emitted from luciferin conversion to oxyluciferin. Here, the pronounced luminescence was recorded for all DAR1-4 types in HTLA cells treated with IBMCat and its enantiomers (Figure 8).

In the case of DAR1, IBMCat and its enantiomers acted as agonists, as expressed by an increase in relative luminescence units (RLU) along with an increasing drug concentration. From a 10-nM concentration of IBMCat, no further increase in RLU was detected, which was probably due to reaching the maximum effective concentration. For DAR2, a significant increase in RLU was observed only at the highest concentration (500 nM) of IBMCat and IBMCat1 (*S*-enantiomer) tested, with an RLU value approximately 5-fold and 3-fold greater than that of the control (transfected but untreated HTLA cells), respectively. Similar results were measured for DAR3, for which a gradual increase in RLU was monitored after IBMCat and IBMCat1 cell treatment. At the highest concentration (500 nM), a 5- and 6.5-fold increase in RLU compared to the control was achieved. At the same time, no effect of the IBMCat2 (*R*-enantiomer) was detected for DAR2 and DAR3-expressing cells. The use of higher concentrations (>1 µM), to better display the dose–response relationship was outweighed by the strong cytotoxicity of IBMCat, therefore, higher concentrations could not be evaluated. The last type of DAR measured was DAR4, in the case of which, there was a maximum 2-fold increase in RLU for cells treated with IBMCat and IBMCat1 and a maximum 1.5-fold increase in RLU for IBMCat2 when compared to untreated control. The results show that IBMCat and both enantiomers are probably the ligands of DAR1 and DAR4. In contrast, for DAR2 and DAR3, only IBMCat and IBMCat1 acted as their ligands.

It is known that DAR1 belongs to a different group of DAR than DAR2, DAR3, and DAR4, which are manifested not only by different sites of occurrence in the organism, but also by different ways of signal induction, and different functions. DAR1 receptors induce a signal by activating adenylate cyclase, whereas DAR2 receptors inhibit the adenylate cyclase activity [39]. Individual DARs in these groups (DAR1-like, DAR2-like) also differ based on the localization of their genes on chromosomes and also the structural similarity. For example, the sequence homology between DAR2 and DAR3 was found to be 75%, and between DAR2 and DAR4 it was only 53% [40]. Differences were also reported for the DA affinity to individual receptor types, with DAR2-like family receptors showing 10-100-fold higher affinity for DA than the DAR1-like family of receptors [41]. Therefore, we assume that IBMCat and its enantiomers may have different affinities for different types of DAR as previously described also for, e.g., 3,4-methylenedioxypyrovalerone [36].

Another group of receptors for which the agonistic effect of IBMCat and its enantiomers was measured was the group of α2 and β adrenergic receptors (ADRA2A, ADRA2B, ADRA2C, and ADRB1, ADRB2, respectively). The agonistic effect was determined in the case of ADRA2B and ADRA2C only in the case of the racemate IBMCat at 500 nM concentration, at which a 1.5 and 3.7-fold increase in RLU was detected when compared to untreated control (ESI, Figure S12). A slight increase in the luminescence signal was also measured for both types of the β adrenergic receptors, i.e., a 3-fold increase in the signal for ADRB1 treated with 500-nM IBMCat, and a 1.5-fold increase for ADRB2 treated with 50 and 100-nM IBMCat and 1 and 5 nM IBMCat1. It is known that the individual groups of α2 and β adrenergic receptors have different effects on the body. While α2 adrenergic receptors inhibit adenylate cyclase and reduce intracellular cyclic adenosine monophosphate (cAMP), activation of β adrenergic receptors, in turn, leads to activation of adenylate cyclase and increased cAMP production. The affinity of the natural ligands adrenaline and noradrenaline for adrenergic receptors is also different, with noradrenaline having a higher affinity for the α-adrenergic and adrenaline for the β-adrenergic receptors. It is therefore not surprising that the prepared cathinone interacts with different receptor subtypes at different levels, as our study shows. At the same time, the prepared cathinone did not exhibit any agonistic activity for HTR2A and HTR2B (ESI, Figure S13). HTR2A-binding activity has been previously described, for example, for mephedrone, flephedrone, and methcathinone. In contrast, HTR receptor binding activity has not been demonstrated for 3,4-methylenedioxymethamphetamine (MDMA) or (3,4-methylenedioxy-*N*-ethylamphetamine) MDEA [37]. Slight activity has been reported for HTR2C.

## 3. Materials and Methods

### 3.1. Chemicals

Solvents used for synthesis (dichloromethane, ethyl acetate) were purchased from Lach-ner s.r.o. Tetrahydrofuran was dried with sodium and freshly distilled before use in the presence of benzophenone. Acetone was dried with calcium(II)chloride, distilled from phosphorus(V)oxide, and stored above molecular sieves. Chemicals used for synthesis were purchased from Merck KGaA (Darmstadt, Germany) and Fluorochem Ltd. (Hadfield, UK). Deuterated methanol (MeOD-*d*_4_, min. 99.8% D) for vibrational circular dichroism (VCD) analysis was purchased from ISOSAR GmbH (Saarbrücken, Germany) and methanol for electron circular dichroism (ECD) analysis with the purity of 99.8% from Sigma-Aldrich Inc. (St. Louis, MO, USA). Hexane, heptane, and propan-2-ol (2-PrOH) for liquid chromatography were purchased from Honeywell (Charlotte, NC, USA), ethanol (EtOH) was obtained from Fischer Scientific (Hampton, NY, USA), and diethylamine (DEA) from Merck KGaA (Darmstadt, Germany).

### 3.2. NMR and MS Analysis

NMR data were acquired on Agilent 400-MR DDR2 spectrometer operating at 400.13 and 100.62 MHz for ^1^H and ^13^C, respectively, using deuterated solvents (chloroform and methanol), purchased from Merck KGaA (Darmstadt, Germany). Mass spectra were obtained by Thermo Scientific LTQ Orbitrap Velos—Hybrid Mass Spectrometer Ultimate Confidence (Bremen, Germany), typically in positive electrospray ionization with methanol as sample diluent.

### 3.3. Chiral Separation

Samples for separation were prepared by the following procedure: the drug sample (1 mg) was dissolved in a mixture of propan-2-ol containing 1% of diethylamine (0.2 mL in total) and topped up to 1 mL with heptane (0.8 mL). The chiral separation in the analytical mode was performed by the Agilent 1100 series system (Agilent Technologies, Waldbronn, Germany) consisting of a solvent tray, degasser, binary pump, an autosampler, and a UV-VIS detector. The flow rate was 1 mL·min^−1^, the temperature was set by a laboratory air conditioning system to 23 °C, the injection volume was 10 μL, and the detection of analytes was performed at λ of 254 nm. The columns used in the analytical screening were polysaccharide-based columns YMC ChiralArt Amylose-SA and YMC ChiralArt Amylose-C, both 250 × 4.6 mm, id, 5 µm (YMC Europe GmbH, Dinslaken, Germany). The screening and optimization of the mobile phase composition using hexane/heptane as the bulk mobile phase with EtOH/2-PrOH as polar constituents and DEA as a basic additive are summarized in Electronic Supplementary Information (ESI).

Chiral separation in preparative mode was performed on Auto-Purification System (Waters, Milford, MA, USA) using polysaccharide-based column Chiralpak IA (250 × 20 mm, id, 5 µm) purchased from Chiral Technologies Europe (Illkirch, France). Based on the screening performed on the analytical scale, the optimum method for the separation of IBMCat was based on a mobile phase consisting of a heptane/2-PrOH (9/1, *v*/*v*) mixture with 0.1% of DEA as a basic additive. The flow rate of the mobile phase was 15 mL·min^−1^, the column temperature was set to 15 °C, and the detection wavelength to 254 nm. The sample concentration of IBMCat was 8 mg·mL^−1^; the injection volume was 0.5 mL.

### 3.4. Chiroptical Methods and DFT Calculations

To obtain VCD and infrared (IR) spectra, the FTIR IFS 66/S spectrometer equipped with a VCD/IRRAS PMA 37 module (Bruker, Bremen, Germany), a ZnSe beam splitter (Hinds Instruments, Hillsboro, OR, USA), a BaF_2_ polarizer, and an MCT detector (InfraRed Associates, Stuart, FL, USA) was used [42]. Individual enantiomers of 4-isobutylmethcathinone hydrochlorides in MeOD at a concentration of 100 g∙L^−1^ were placed into BioCell cuvette with CaF_2_ windows and an optical path length of 27.3 mm (BioTools, Inc., Jupiter, FL, USA). The VCD spectra were recorded with a resolution of 8 cm^−1^ at ambient temperature in the spectral range of 1800–1250 cm^−1^ and averaged from 12 blocks to enhance the signal to noise. Each block was measured for 20 min and contained 3680 interferometric records. The IR spectra were obtained as the accumulation of 32 scans. The VCD and IR baseline was corrected by the subtraction of the solvent (D_2_O) spectra measured under the same experimental conditions to obtain the final VCD and IR spectra.

The experimental ECD spectra were recorded using a J-815 spectrometer (JASCO Corporation, Tokyo, Japan) purged with nitrogen gas (purity 99.99%, Siad, Prague, Czech Republic) during the measurement. The individual solutions of 4-isobutylmethcathinone enantiomers in MeOH (Penta, Prague, Czech Republic) at a final concentration of 333 mg∙L^−1^ were placed into a quartz cuvette with a path length of 1 mm (Hellma, Müllheim, Germany) and measured at ambient temperature in a spectral range of 195–380 nm with following experimental conditions: scanning speed of 20 nm∙min^−1^, a response time of 8 s, and a bandwidth of 1 nm. The ECD spectra were averaged from 3 accumulations and the baseline correction was applied as a subtraction of the ECD spectra of solvent (MeOH) measured under identical experimental conditions. The corresponding UV absorption spectra were calculated from the HT voltage detector using the Spectra Manager software (JASCO Corporation, Tokyo, Japan), specifically the Spectra Analysis module.

**Figure 9:**
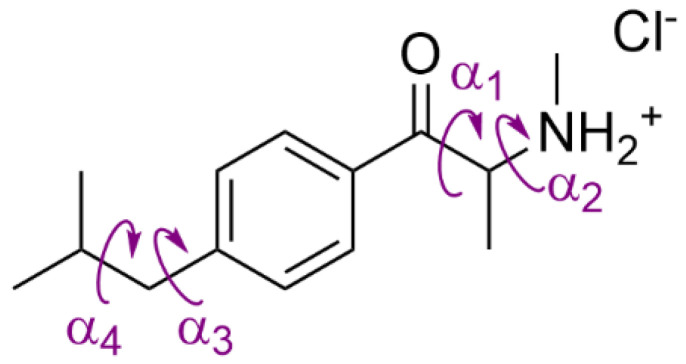
Dihedral angles α_1_, α_2_, α_3_ and α_4_ of 4-isobutylmethcathinone.

For DFT calculations of stable conformers of (*S*)-4-isobutylmethcathinone, the starting geometries were generated by the systematic rotation of four dihedral angles α_1_, α_2_, α_3,_ and α_4_ (Figure 9), each with a step of 120° using the MCM software [43]. This approach yielded 81 starting geometries that were subsequently optimized at the B3LYP/6-31G(d) level of theory. After this optimization, only 5 stable conformers of (*S*)-4-isobutylmethcathinone within 4 kcal∙mol^−1^ were revealed and further optimized at a higher B3LYP/6-311++G(d,p) level of theory. The solvent effect of methanol was considered via a conductor-like polarizable continuum model with dielectric constant *ε* = 32.613. The relative abundances of the stable conformers were calculated using the Boltzmann distribution based on the Gibbs free energies (*T* = 273 K).

To simulate the hydrogen-deuterium exchange in the VCD and IR absorption spectra in MeOD, the two exchangeable hydrogen atoms of the NH_2_^+^ group were replaced by deuterium isotopes in the DFT calculations. The VCD and IR bands were visualized using a Lorentzian function with a 10 cm^−1^ half-width at half maximum.

To simulate the ECD and UV absorption spectra, time-dependent (TD) DFT calculations at the TD-B3LYP/6-311++G(d,p) level of theory with the 30 lowest singlet states of each conformer were used and the Gaussian profile with a bandwidth of 20 nm was applied.

The optimum scaling factors for simulated spectra were evaluated using the CDSpecTech program package based on the Tanimoto coefficient [44,45].

### 3.5. Cell Lines and Cultivation

In this study, the following cell lines were used: HEK 293T, Hep G2, HMC-3, SH-SY5Y, 5637, CaCo-2, HaCaT, MRC-5, BJ, and L929. The cells lines were of the following origin: human embryonic immortalized kidney cells (HEK 293T), cells from hepatocellular carcinoma (Hep G2), microglial immortalized cells (HMC-3), cells from neuroblastoma (SH-SY5Y), the urinary bladder carcinoma (5637), colon carcinoma (CaCo-2), immortalized keratinocytes (HaCaT), noncancerous lung fibroblasts (MRC-5), fibroblasts from the foreskin (BJ) and mouse immortalized fibroblasts (L929; frequently used for cytotoxicity evaluation). The selected cell lines were chosen so that not only primary noncancerous cells are evaluated but also cells derived from tissues, which are the most often affected by novel synthetic drugs, i.e., kidney, liver, urinary bladder, and nervous tissue. Except for L929 and MRC-5 which were supplied by Sigma-Aldrich, the rest of the cell lines were purchased from the American Tissue Culture Collection (ATCC). The cells were maintained as recommended by the ATCC, i.e., at the exponential phase of growth in the recommended cultivation media supplemented with stable L-glutamine and 10% fetal bovine serum (Thermo Fisher Scientific, Waltham, MA, USA), except for CaCo-2 cells, for which 20% fetal bovine serum is needed. The cells were handled and cultivated in sterile conditions at 37 °C, 5% CO_2_, and 95% humidity. The cells were routinely morphologically examined and tested for mycoplasma.

### 3.6. Cytotoxicity Tests

The cytotoxicity of IBMCat was evaluated in vitro by using a WST-1 viability assay (Sigma-Aldrich, Saint Louis, MO, USA) based on the transformation of a tetrazolium salt into colored formazan in metabolically active cells. A total of 5000 cells (or 10,000 for MRC-5 and BJ cells) per well of 96-well plate were seeded in 100 µL of complete cell cultivation media and kept in the incubator for the following 16 h. Then, the cells were treated with IBMCat and its enantiomers at a concentration range of 100 µL of cell cultivation media, which was added to the 100 µL of media already contained in the wells. After 72 h of additional incubation of the cells, their viability was determined by WST-1. Briefly, the culture medium was discarded and changed for 100 µL of phenol red-free Dulbecco’s Modified Eagle Medium (DMEM) with the addition of 5 µL of the WST-1 reagent. The cells were incubated for 2 h, after which absorbance of the raised formazan was measured spectrophotometrically at 450 nm (reference wavelength of 630 nm) using a UV-Vis spectrometer (Bio-Rad Laboratories, Hercules, CA, USA). The samples were prepared in quadruplicates. Cells treated only with a vehicle and untreated cells served as controls. The half-maximal inhibitory concentrations (IC_50_) were calculated from the measured dose–response curves by AAT Bioquest.

### 3.7. Fluorescence Microscopy and Sample Preparation

A total of of 1 × 10^5^ CaCo-2 and MRC-5 cells were seeded into 35-mm glass-bottom dishes for live-cell imaging ((#1.5, MatTek, USA; Ibidi dishes, Germany). After 16 h, the cells were gently washed with phosphate-buffered saline (PBS; pH 7.4), and then, cultivation media containing 100–500 µM IBMCat was added. Untreated cells and cells treated with the vehicle served as controls. After 24 h of the cell incubation with IBMCat, the cells were gently washed with PBS and then fixed with a 4% solution of formaldehyde (methanol-free, Thermo Fisher, USA) in PBS for 20 min. at 22 °C in the dark. Then, the fixative was discarded, and the cells were washed twice with PBS. The cell structures were stained with a phalloidin-Atto 488 (Atto-tec, Siegen, Germany) solution and 4’,6-diamidino-2-phenylindole (Sigma-Aldrich, USA) for 15 min. to stain F-actin and cell nuclei, respectively. After that, the cells were washed twice with PBS and subjected to fluorescence microscopy. The impact of IBMCat on cell morphology was monitored by Olympus IX-81 wide-field fluorescence microscope (Olympus, Tokyo, Japan). The images were acquired by EM-CCD camera C9100-02 (Hamamatsu, Hamatsu, Japan) using xCellence software, 60× oil immersion objective (NA of 1.4), and a high-stability 150 W xenon burner. The images were background-corrected, and the individual channels were merged, both using xCellence software (Olympus, Tokyo, Japan).

### 3.8. PRESTO-Tango β-Arrestin Recruitment Assay

The HTLA cell line was used for the PRESTO-Tango β-arrestin recruitment assay [38]. The HTLA cells were provided as a gift from the laboratory of Bryan Roth (Roth Lab, USA). It is a human cell line derived from human embryonic kidney cells (HEK 293) stably expressing β-arrestin in a fusion with the tobacco etch virus protease (TEV) protease and the luciferase gene under the control of the transcription factor tetracycline transcriptional activator (tTA) [38]. The HTLA cells were cultured under standard incubation conditions (37 °C, 5% CO_2_, 95% humidity) in DMEM with 10% FBS. To study the interaction of the prepared substances with the dopamine, adrenergic, and serotonin receptors, the HTLA cells were detached from the surface of the cell culture dish with 0.25% trypsin-ethylenediaminetetraacetic acid solution and seeded at the density of 1 × 10^6^ cells per culture dish with the diameter of 60 mm in 5 mL of DMEM supplemented with 10% FBS. After 24 h of cultivation, the cells were transfected with 2 µg of plasmid DNA using JetOPTIMUS transfection reagent (Polyplus, Berkeley, CA, USA), according to the manufacturer’s protocol.

The transfection was performed by using plasmid DNA for individual types of the dopamine, adrenergic, and serotonin receptors in a fusion with the Tango construct (Table S3).

The transfected HTLA cells were incubated for 24 h, and then 1 × 10^4^ cells were seeded per well of a white 96-well clear bottom plate (Brand, Germany) in 40 μL of DMEM with 1% charcoal-stripped FBS (Thermo Fisher Scientific, USA). After 6 h of incubation, the cells were treated with 5× concentrated test substance solution (10 µL per well) diluted in DMEM with 1% charcoal-stripped FBS. The final concentrations of the solutions in the wells were 0.5, 1, 5, 10, 50, 100, and 500 nM. Transfected cells treated with dopamine, serotonin, and noradrenaline as well as untreated (transfected) cells served as controls. After the treatment, the cells were further cultured for 20 h under standard cultivation conditions. Then, 50 μL of pre-prepared OneGlo reagent (Promega, Madison, WI, USA) containing a luciferase substrate and lysis reagent was added to the wells. After 5 min., the luminescence was measured using a multi-mode microplate reader SpectraMax iD5 (Molecular Devices, San Jose, CA, USA) measuring the entire wavelength spectrum. All samples were measured in quadruplicates. The data were evaluated by Prism 7 software (GraphPad, San Jose, CA, USA).

### 3.9. Data Analysis

The data are represented as the mean value of four replicates plus minus the standard error of the mean. The data analysis was performed by one-way analysis of variance (ANOVA) test using GraphPad Prism software v. 7 (San Diego, CA, USA).

## 4. Conclusions

Here, we present the synthesis of a novel cathinone bearing an isobutyl chain in position four of the aromatic unit. We elaborated on the chiral separation of the individual enantiomers of this chiral drug on an analytical as well as preparative scale. Subsequently, we characterized the enantiomers spectroscopically using chiroptical methods supported by quantum chemical calculations, which enabled us to determine their absolute configuration. The individual enantiomers and the racemic mixture were then subjected to an in vitro cytotoxicity study, and the results were compared with those reported in the literature for other synthetic cathinones. We found that the new synthetic cathinone is substantially more toxic than its structurally related substances. On the other hand, chiral toxicity, dependent on the absolute configuration of the enantiomers, was not observed. In the receptor binding studies, we have observed not only strong differences among the IBMCat enantiomers but also different behavior of the drug in comparison to synthetic cathinones of similar structure. The behavior of IMBCat is more closely related to methylenedioxy-bridged substances, such as MDPV and MDMA. The combination of high toxicity, particularly hepatotoxicity and neurotoxicity, with the receptor-binding properties of MDPV makes 4-isobutylmethcathinone dangerous for human health and qualifies it as a serious threat to society. Due to its high similarity to already existing synthetic cathinones, it is expected to appear rather soon on the market and, therefore, the legal authorities are kindly asked to list the substance as controlled.

## Figures and Tables

**Table 1:** Structural parameters of the most stable conformers of 4-isobutylcathinone: the values of dihedral angles α_1_, α_2_, α_3,_ and α_4_, relative Gibbs free energies, and relative abundances based on the Boltzmann distribution. The calculations were performed at the B3LYP/6-311++G(d,p) level.

	Dihedral Angles					
Conformer	α_1_	α_2_	α_3_	α_4_	Δ*G* (kJ mol^−1^)	Relative Abundances (%)
4-isobutylmethcathinone I	96	−80	−75	−64	0	38
4-isobutylmethcathinone II	97	−80	108	173	0.23	25
4-isobutylmethcathinone III	97	−80	−105	−173	0.37	20
4-isobutylmethcathinone IV	97	−81	74	−173	0.54	15
4-isobutylmethcathinone V	97	−81	89	63	1.91	2

**Table 2:** Evaluation of in vitro cytotoxicity of 4-isobutylmethcathinone (IBMCat) and its enantiomers after 72 h of incubation with individual cell lines. IC_50_—half maximal inhibitory concentration, i.e., a concentration of a compound required to achieve a 50% reduction in cell viability; SD—standard deviation (from four replicates).

Compound	IBMcat	S-IBMCat	*R*-IBMCat
Cell Line			
	IC_50_ ± SD [µM]		
HEK 293T	123 ± 6	192 ± 2	185 ± 5
Hep G2	52 ± 7	46 ± 9	49 ± 1
HMC-3	61 ± 2	64 ± 1	65 ± 2
SH-SY5Y	44 ± 5	50 ± 1	52 ± 4
5637	18 ± 1	21 ± 4	20 ± 1
L929	36 ± 4	33 ± 5	39 ± 2
CaCo-2	389 ± 42	390 ± 17	331 ± 19
HaCaT	357 ± 40	361 ± 22	329 ± 43
MRC-5	635 ± 17	530 ± 35	515 ± 34
BJ	749 ± 102	685 ± 41	698 ± 88

## Data Availability

Data is contained within the article and supplementary material.

## Supplementary Materials

The following supporting information can be downloaded at: https://www.mdpi.com/article/10.3390/ph15121495/s1, Scheme S1: General scheme of synthesis of 4-isobutylmethcathinone (4) and its formal metabolite; Figure S1: Mass spectrum of compound 4; Figure S2: Mass spectrum of compound 5; Figure S3: Mass spectrum of compound 6; Figure S4: HPLC chromatograms of IBMCat in heptane (red) and hexane (green) mobile phases measured on Amylose-SA column, and heptane (blue) and hexane (orange) mobile phases measured on Amylose-C column; Figure S5: Chromatograms of IBMCat in heptane (red) and hexane (green) mobile phases measured on Amylose-SA; Figure S6: Chromatograms of IBMCat in heptane (purple) and hexane (grey) mobile phases with ethanol as polar modifier measured on column Amylose-SA; Figure S7: Chemical structure of chiral strong cation-exchange sorbents in CSP I and CSP II; Figure S8: Chromatogram of IBMCat on cation-exchange columns CSP I (blue) and CSP II (green); Figure S9: The structures of the stable conformers II, III, IV and V of 4-isobutylmethcathinone hydrochloride in MeOH simulated at B3LYP/6-311++G(d,p)/CPCM level; Figure S10: Comparison of the IR absorption spectra of enantiomers of IBMCat in MeOD-*d*_4_: the spectrum simulated at the B3LYP/6-311++G(d,p) level (top) and experimental spectra (bottom); Figure S11: Comparison of the UV absorption spectra of enantiomers of IBMCat in MeOH: the simulated spectrum at the TD-B3LYP/6-311++G(d,p) level (top) and experimental spectra (bottom); Figure S12: β-arrestin 2 recruitment PRESTO-Tango assay: activation of the adrenergic receptors; Figure S13: β-arrestin 2 recruitment PRESTO-Tango assay: activation of the serotonin receptors; Table S1: Retention times and calculated chromatographic parameters of IBMCat for each mobile phase employed on columns Amylose-SA (red and green) and Amylose-C (blue and orange); Table S2: Retention times and calculated analytical parameters of IBMCat for mobile phases containing either propan-2-ol or ethanol as modifier; column—Amylose-SA; Table S3: Plasmid DNA and its source used in PRESTO-Tango β-arrestin recruitment assay. References [28,46,47,48,49,50] are cited in the Supplemmtary Materials.
