# Supplementary material for: 4-Isobutylmethcathinone—A Novel Synthetic Cathinone with High In Vitro Cytotoxicity and Strong Receptor Binding Preference of Enantiomers
Source: Pharmaceuticals (Basel). 2022 Nov 30;15(12):1495. doi: 10.3390/ph15121495 (PMC9780888; doi:10.3390/ph15121495)

Electronic supplementary information

4-Isobutylmethcathinone – A novel synthetic cathinone with high *in vitro* cytotoxicity and strong receptor binding preference of enantiomers

Martin Paškan[a], Silvie Rimpelová[b], Vladimíra Svobodová Pavlíčková[b], Dita Spálovská[c] Vladimír Setnička[c], Martin Kuchař[d], Michal Kohout[a*]

[a] Department of Organic Chemistry, University of Chemistry and Technology Prague, The Czech Republic

[b] Department of Biochemistry and Microbiology, University of Chemistry and Technology Prague, The Czech Republic

[c] Department of Analytical Chemistry, University of Chemistry and Technology Prague, The Czech Republic

[d] Forensic Laboratory of Biologically Active Substances, Department of Chemistry of Natural Compounds, University of Chemistry and Technology, Prague 6, Czech Republic

1. Synthesis and characterization

The synthesis of 4-isobutylmethcathinone started with Friedel-Crafts acylation to form ketone **2** [46], which was subjected to α-bromination via copper(II) bromide in ethyl acetate to form intermediate **3** [47]. To form the target substance **4**, ketone **3** undergoes nucleophilic substitution with methylamine in tetrahydrofuran. Precursor **3** can further be used to prepare 4-isobutylcathinone (**6**), which can be considered as a formal metabolite of the target drug **4**. To synthesize **6**, the bromoketone **3** reacted with sodium azide in dry acetone to form azide **5**. This material was reduced via catalytic hydrogenation on palladium giving rise to 4-isobutylcathinone **6**. To stabilize the prepared compounds and avoid their degradation (e.g., via intramolecular cyclization [28]), both final products (**4** and **6**) were transformed to hydrochloric salts.

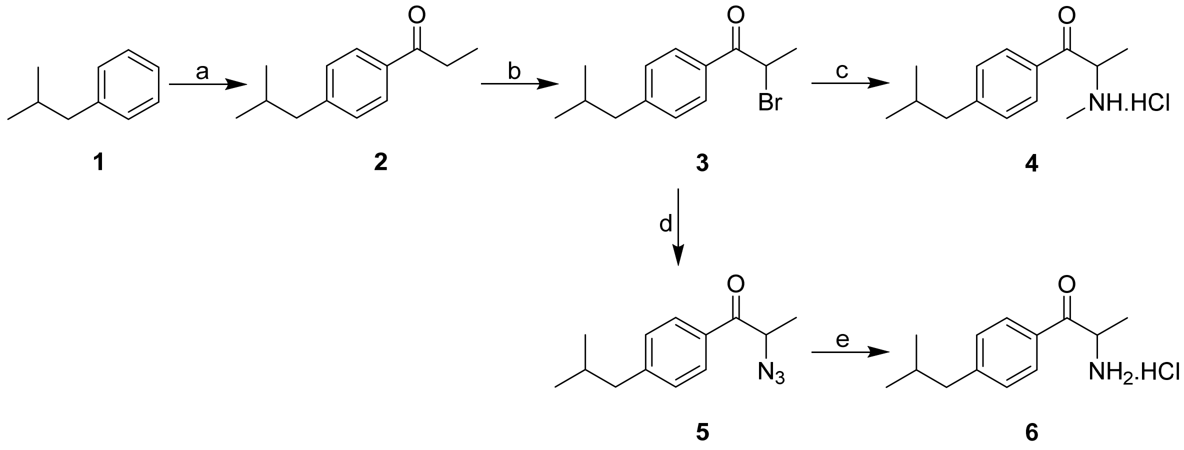

Reagents: a) propionyl chloride, $AlCl_3$, CH_2Cl_2; b) $CuBr_2$, EtOAc; c) NH_2CH_3, THF, HCl; d) NaN_3, AcOH; e) H_2, Pd/C, MeOH, HCl;

Scheme S1: *General scheme of synthesis of 4-isobutylmethcathinone (4) and its formal metabolite.*

Experimental section:

1-(4-Isobutylphenyl)propan-1-one (2)

Propionyl chloride (7.57 g; 81.8 mmol) was added to a suspension of $AlCl_3$ (10.9 g; 81.8 mmol) in CH_2Cl_2 (100 ml) at 0 °C and the reaction mixture was stirred for 30 min at this temperature, then, isobutylbenzene (10.2 g; 76.2 mmol) was added. After stirring for 1 h at 0 °C, the reaction mixture was poured into crushed ice. The organic layer was separated and washed with water (2 x 50 mL), brine (50 mL) and dried with $MgSO_4$. Dichloromethane was evaporated and the product was distilled under pressure (100 °C/0.312 kPa) to give **2** (10.5 g; 68%) as a yellow oil. ^{1}H NMR ($CDCl_3$) (Appendix 1 for full spectrum): ^{1}H NMR ($CDCl_3$): 0.90 (d, 6H, CH_3, J = 6.73); 1.22 (t, 2H, CH_3, J = 7.28); 1.89 (m, 1H, CH); 2.52 (d, 2H, CH_2, J = 7.25); 2.98 (q, 2H, CH, J = 7.25); 7.21 (d, 2H, CH, J = 8.28); 7.88 (d, 2H, CH, J = 8.25) ^{13}C NMR ($CDCl_3$): 8.33 (CH_3); 22.34 (CH_3, CH_3 – iBu); 30.08 (CH, CH – iBu); 31.53 (CH_2); 45.30 (CH_2, CH_2 – iBu); 127.91 (CH, CH – Ar); 129.20 (CH, CH – Ar); 134.66 (C, C – Ar); 147.17 (C, C – Ar); 200.26 (C, CO). The spectroscopic data of compound **2** was similar to the literature [48]: IR (neat): 2955, 1682, 1607, 1460, 1225, 1180, 951, 783 cm^{-1}; HRMS (NSI): $[M+H]^+$ calc. 191.1430, found 191.1426 $[C_{13}H_{19}O]^+$

2-Bromo-1-(4-isobutylphenyl)propan-1-one (3)

Ketone **2** (0.250 g; 1.31 mmol) was dissolved in ethyl acetate (7 mL), then, copper (II) bromide (0.443 g; 1.98 mmol) was added. The reaction mixture was refluxed for 4 h. Subsequently, the reaction mixture was washed with water (3 x 10 mL), brine (10 mL), and dried with $MgSO_4$. Ethyl acetate was evaporated and the residue was purified using column chromatography to form α-bromo-derivative **3** (0.3 g; 85 %), white crystals. M.p. 63-64.2 °C (lit. 65-66 °C [5]). ^{1}H NMR ($CDCl_3$): 0.90 (d, 6H, CH_3, J = 6.6); 1.90 (m, 4H, CH, CH_3); 2.54 (d, 3H, CH_3, J = 7.2); 5.29 (q, 1H, CH); 7.25 (d, 2H, CH, J = 8.4); 7.94 (d, 2H, CH, J = 8.1) ^{13}C NMR ($CDCl_3$): 20.21 (CH_3); 22.37 (CH_3, CH_3 – iBu); 30.10 (CH, CH – iBu); 41.54 (CH_2); 45.43 (CH_2, CH_2 – iBu); 128.93 (CH, CH – Ar); 129.49 (CH, CH – Ar); 133.69 (C, C – Ar);146.41(C, C – Ar); 193.06 (C, CO). The spectroscopic data of compound **3** was similar to the literature [49] IR: 2955, 1690, 1610, 1420, 1265, 1255, 1160, 955, 865 cm^{-1}; MS: m/z 270 (M^+, 3), 268 (M^+, 4), 161 (100), 147 (8), 131 (7), 118 (18), 105 (7), 91 (21), 77 (8).

1-(4-Isobutylphenyl)-2-(methylamino)propan-1-one hydrochloride (4)

Bromo-derivative **3** (3 g; 11.1 mmol) was dissolved in THF (15 mL) and methylamine (2.5 mL; 66.6 mmol) was added. The reaction mixture was stirred for 4 h under inert atmosphere. Then, water (10 mL) was added and the solution was stirred for another 10 min. The reaction mixture was extracted with ethyl acetate (3 x 15 mL). The combined organic solution was washed with brine (15 mL) and dried with $MgSO_4$ in a fridge. The drying agent was filtered off, and the solvent was evaporated to the volume of 4 mL, which was treated with hydrochloric acid (1 mL; 35 %). The mixture was evaporated giving rise to 4-isobutylmethcathinone (**4**) (2.1 g; 86%)

as a white solid. M.p. 216-217.2 °C. ^{1}H NMR (CH_3OD): 0.92 (d, 6H, CH_3, J = 6.8); 1.57 (d, 3H, CH_3, J = 7.2); 1.93 (m, 1H, CH); 2.60 (d, 2H, CH_2, J = 7.2); 2.76 (s, 3H, CH_3); 5.06 (q, 1H, CH); 7.40 (d, 2H, CH, J = 8.8); 7.97 (d, 2H, CH, J = 8.1) ^{13}C NMR (CH_3OD): 15.52 (CH_3); 21.22 (CH_3, CH_3 – iBu); 29.93 (CH, CH – iBu); 30.20 (CH_3, N – CH_3); 40.98 (CH2, CH2 – iBu); 58.12 (CH); 128.58 (CH, C – Ar); 129.66 (CH, C – Ar); 131.14 (C, C – Ar); 151.48 (C, C – Ar); 195.11 (C, CO). HRMS-ESI: m/z calculated for [$C_{14}H_{21}NO$] + H^+ $[M+H]^+$ 220.17, found 220.16956 (Fig. S1).

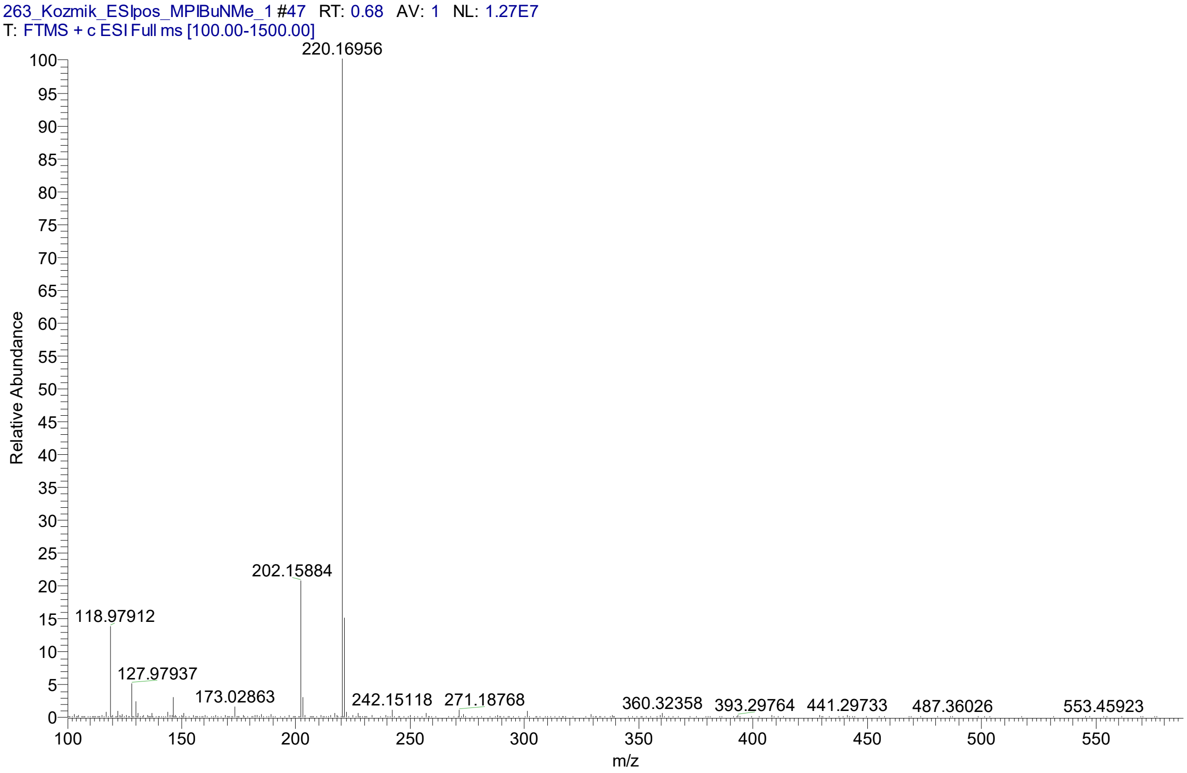

Figure S1: Mass spectrum of compound **4**.

2-Azido-1-(4-isobutylphenyl)propan-1-one (5)

Bromo-derivative **3** (1 g; 3.71 mmol) and sodium(I)azide (0.5 g; 7.69 mmol) were stirred in dry acetone (25 mL) under inert atmosphere for 3 h. The reaction mixture was filtered, washed with acetone, and then evaporated. Azide **5** was purified on silica to obtain yellow oil (0.81 g; 94 %). ^{1}H NMR ($CDCl_3$): 0.90 (d, 6H, CH_3, J = 6.8); 1.55 (d, 2H, CH_2, J = 7.2); 1.90 (m, 1H, CH); 2.53 (d, 3H, CH_3, J = 7.2); 4.70 (q, 1H, CH); 7.26 (d, 2H, CH, J = 8.4); 7,85 (d, 2H, CH, J = 8.4) ^{13}C NMR ($CDCl_3$): 16.57 (CH_3); 22.33 (CH_3, CH_3 – iBu); 30.13 (CH, CH – iBu); 45.41 (CH_2); 58.21 (CH_2, CH_2 – iBu); 128.69 (CH, CH – Ar); 129.67 (CH, CH – Ar); 131.91 (C, C – Ar); 148.62 (C, C – Ar); 196.35 (C, CO). HRMS-ESI: m/z calculated for $[C_{13}H_{17}NO]^+$ 203.13; 204.13, found 203.1309; 204.1341 (Fig. S2).

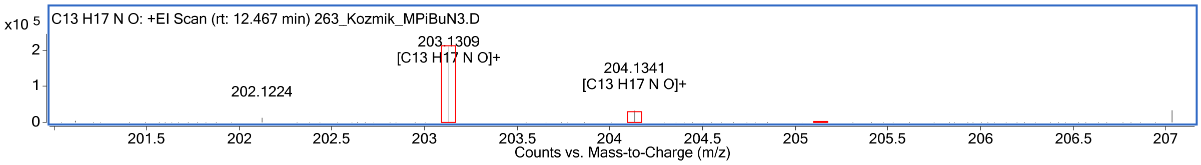

Figure S2: Mass spectrum of compound **5**.

2-Amino-1-(4-isobutylphenyl)propan-1-one hydrochloride (6)

To a solution of azide **5** (0.9 g; 3.8 mmol) in methanol (10 mL), a palladium catalyst (90 mg Pd/C) was added. The reaction mixture was stirred at room temperature in hydrogen atmosphere for 1 h. The reaction was quenched with hydrochloric acid (1 mL; 35 %) and evaporated to obtain white crystals of **6** (0.53 g; 66 %). M.p. 220-221 °C. ^{1}H NMR (CH_3OD): 0.90 (d, 6H, CH_3, J = 6.7); 1.56 (d, 3H, CH_3, J=8); 1.93 (m, 1H, CH); 2.59 (d, 2H, CH_2, J = 7.6); 5.07 (q, 1H, CH); 7.38 (d, 2H, CH, J = 8.7); 7.97 (d, 2H, CH, J = 8) ^{13}C NMR (CH_3OD): 16.42 (CH_3); 21.24 (CH_3, CH_3 – iBu); 29.93 (CH, CH – iBu); 44.95 (CH2, CH2 – iBu); 51.27 (CH); 128.52 (CH, C – Ar); 129.62 (CH, C–Ar); 130.50 (C, C – Ar); 149.50 (C, C – Ar); 195.32 (C, CO). HRMS-ESI: m/z calculated for $[C_{13}H_{19}NO] + H^+$ $[M+H]^+$ 206.15, found 206.15378 (Fig. S3).

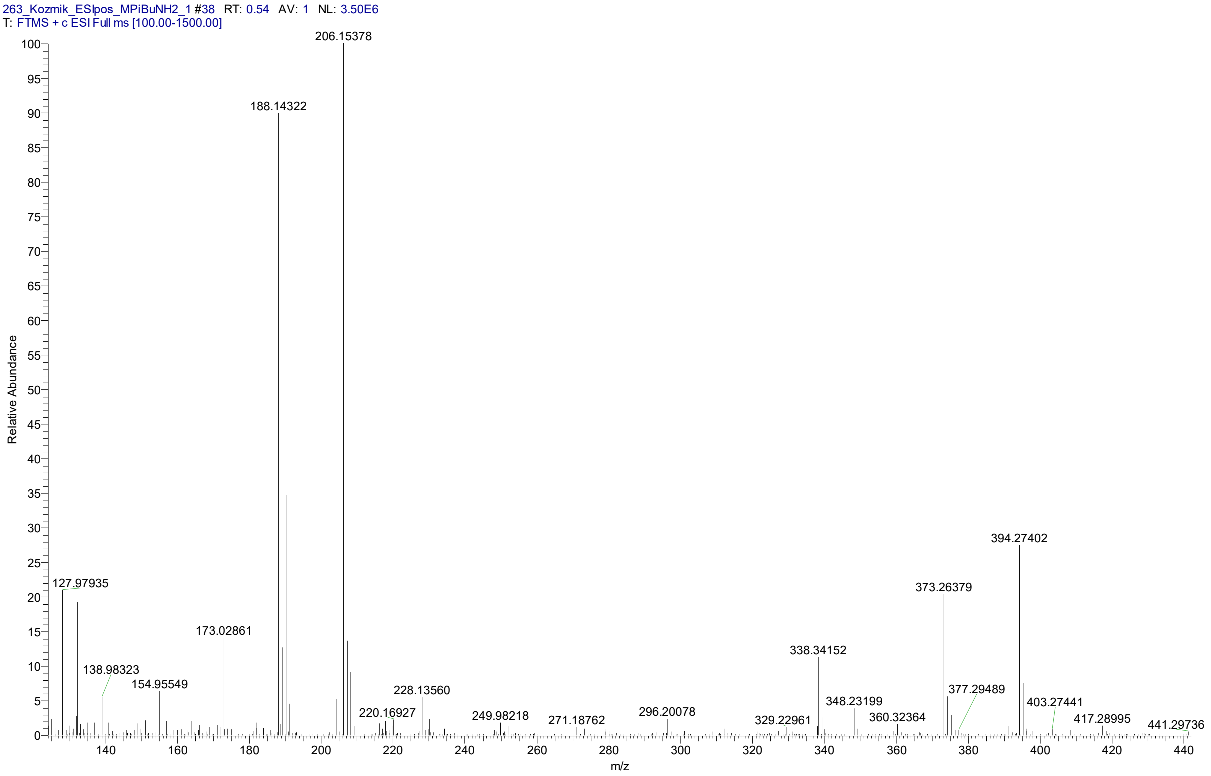

Figure S3: Mass spectrum of compound **6**.

2. Chiral separation

Although the formal metabolite **6** of the IBMCat was not utilized in the biological part of this study, it was subjected to chiral separation. The enantioseparation of compounds **4** and **6** was performed at temperature 25 °C with a flow of 1 mL·min^{-1}, an injection volume of 10 µl and a detection wavelength of 254 nm. Two polysaccharide-based columns differing in surface chemistry, namely immobilized YMC ChiralArt Amylose-SA (150x4.6 mm, 5µm) and coated YMC ChiralArt Amylose-C. were utilized. The ratio of pre-selected bulk solvents and polar modifier (propan-2-ol) was optimized using a constant volume of diethylamine as the base.

Table S1: Retention times and calculated chromatographic parameters of IBMCat for each mobile phase employed on columns Amylose-SA (red and green) and Amylose-C (blue and orange).

Mobile phase	c.c	tr₁ (min)	tr₂ (min)	k₁	k₂	α	R	N1	N2
HEP/IPA 95/5 + 0.1% DEA		7.096	7.743	1.007	1.190	1.182	2.101	10346	11493
HEP/IPA 90/10 + 0.1% DEA		5.559	5.900	0.588	0.686	1.166	1.480	11578	11359
HEP/IPA 85/15 + 0.1% DEA		4.814	5.092	0.368	0.447	1.215	1.376	12008	11151
HEX/IPA 95/5 + 0.1% DEA		6.911	7.843	0.943	1.206	1.278	3.098	12047	12175
HEX/IPA 90/10 + 0.1% DEA		5.382	5.835	0.519	0.647	1.246	2.032	12479	12468
HEX/IPA 85/15 + 0.1% DEA		4.624	4.893	0.314	0.390	1.243	1.409	12694	11760
HEP/IPA 95/5 + 0.1% DEA		6.208	6.970	0.756	0.971	1.285	1.671	4133	4327
HEP/IPA 90/10 + 0.1% DEA		6.155	6.944	0.759	0.984	1.297	1.330	2706	2284
HEP/IPA 85/15 + 0.1% DEA		5.219	5.676	0.484	0.613	1.269	1.161	3724	3941
HEX/IPA 95/5 + 0.1% DEA		6.935	7.892	0.950	1.219	1.283	1.531	2239	3614
HEX/IPA 90/10 + 0.1% DEA		5.469	6.029	0.544	0.702	1.291	1.375	4041	4053
HEX/IPA 85/15 + 0.1% DEA		4.928	5.368	0.400	0.525	1.312	0.897	2191	2349

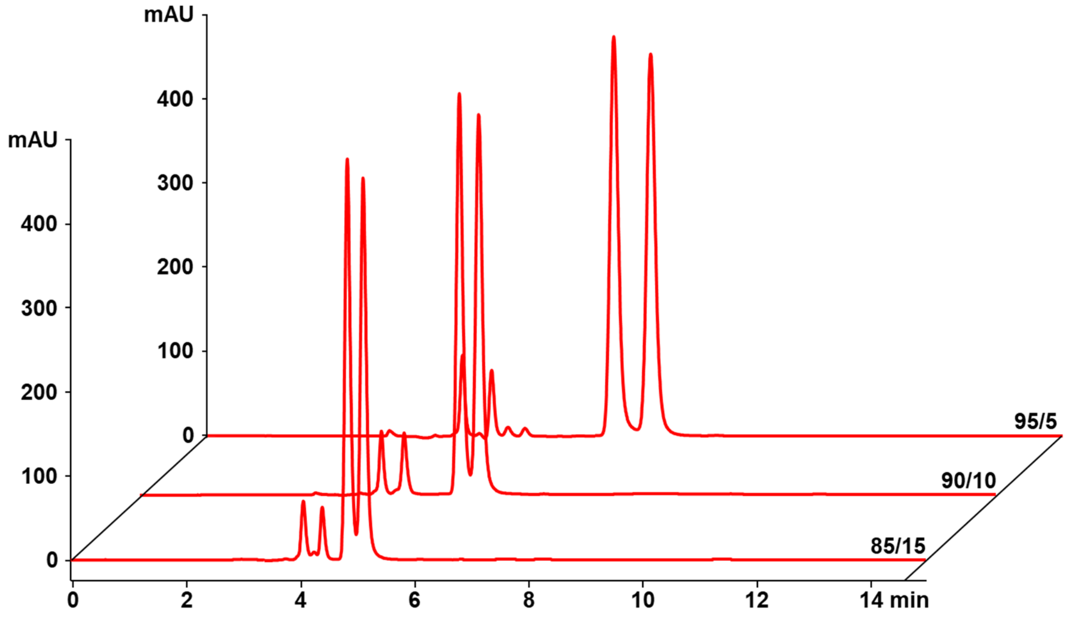

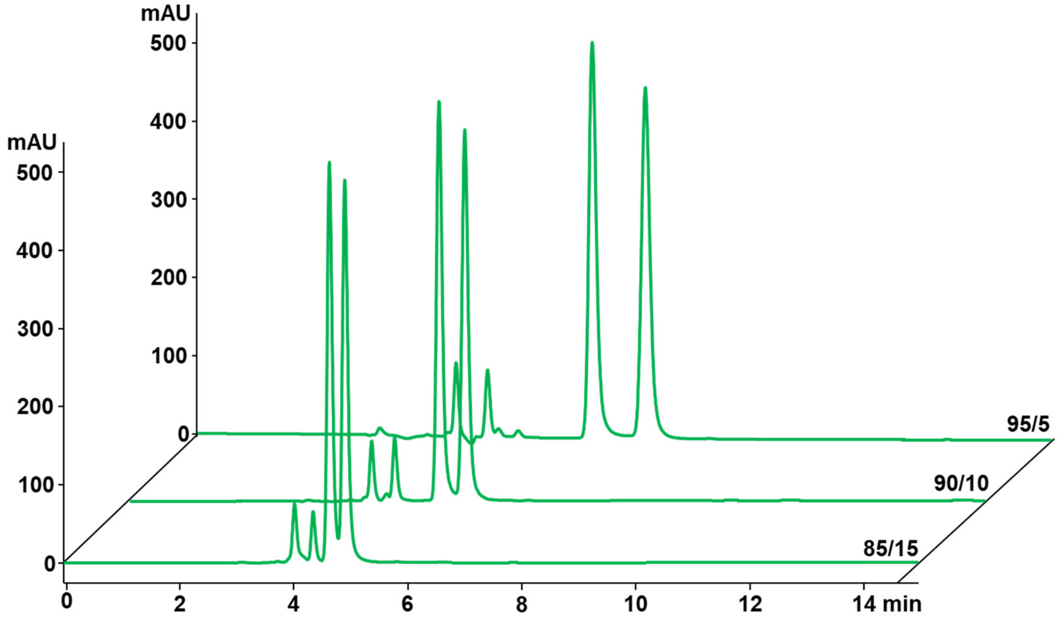

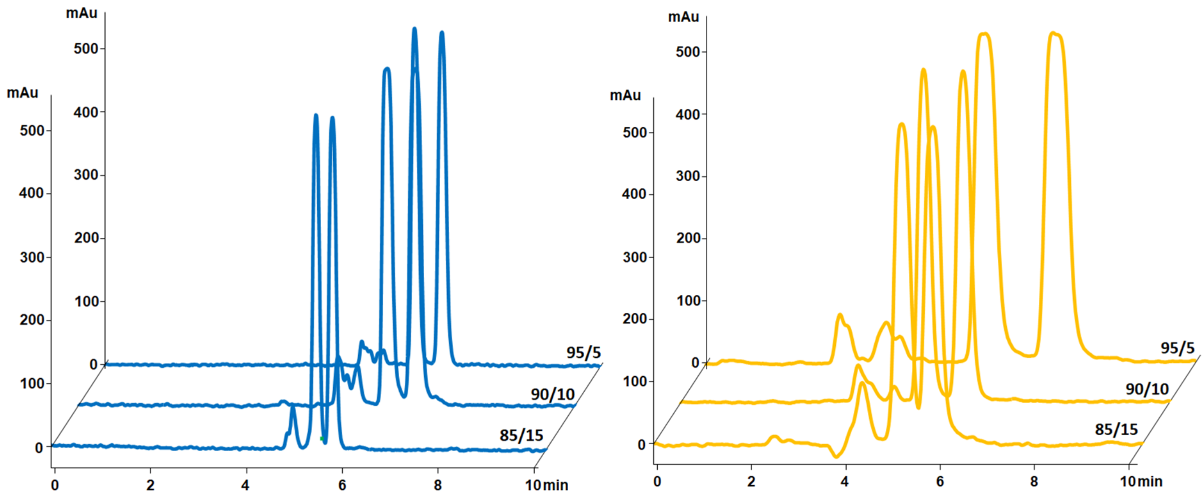

Figure S4: HPLC chromatograms of IBMCat in heptane (red) and hexane (green) mobile phases measured on Amylose-SA column, and heptane (blue) and hexane (orange) mobile phases measured on Amylose-C column.

In the case of formal metabolite, 4-isobutylcathinone, only Amylose-SA column was chosen for separation. Given the higher polarity of this analyte, the polar modifier of the mobile phase was changed from propan-2-ol to ethanol.

Table S2: Retention times and calculated analytical parameters of IBMCat for mobile phases containing either propan-2-ol or ethanol as modifier; column - Amylose-SA.

**Mobile phase**	**c.c**	**tr_1 (min)**	**tr_2 (min)**	**k_1**	**k_2**	**α**	**R**	**N1**	**N2**
HEP/IPA 95/5 + 0.1% DEA		20.22	35.21	4.72	8.96	1.90	5.51	6733	1202
HEP/IPA 90/10 + 0.1% DEA		11.36	18.69	2.25	4.34	1.93	6.99	8501	3839
HEP/IPA 85/15 + 0.1% DEA		8.51	12.33	1.42	2.50	1.77	5.90	9098	5010
HEX/IPA 95/5 + 0.1% DEA		10.97	24.60	2.11	5.97	2.83	11.45	9145	6651
HEX/IPA 90/10 + 0.1% DEA		7.86	15.49	1.17	3.27	2.80	10.07	9481	7189
HEX/IPA 85/15 + 0.1% DEA		21.13	30.45	4.94	7.56	1.53	5.04	7353	2818
HEP/EtOH 9/1 + 0.1% DEA		11.43	16.40	2.23	3.63	1.63	5.92	9928	4586
HEP/EtOH 8/2 + 0.1% DEA		7.96	10.88	1.26	2.09	1.66	5.14	8776	5353
HEX/EtOH 9/1 + 0.1% DEA		13.34	29.31	2.75	7.23	2.63	11.98	8528	8691
HEX/EtOH 8/2 + 0.1% DEA		8.20	16.26	1.33	3.62	2.72	10.84	10319	8303

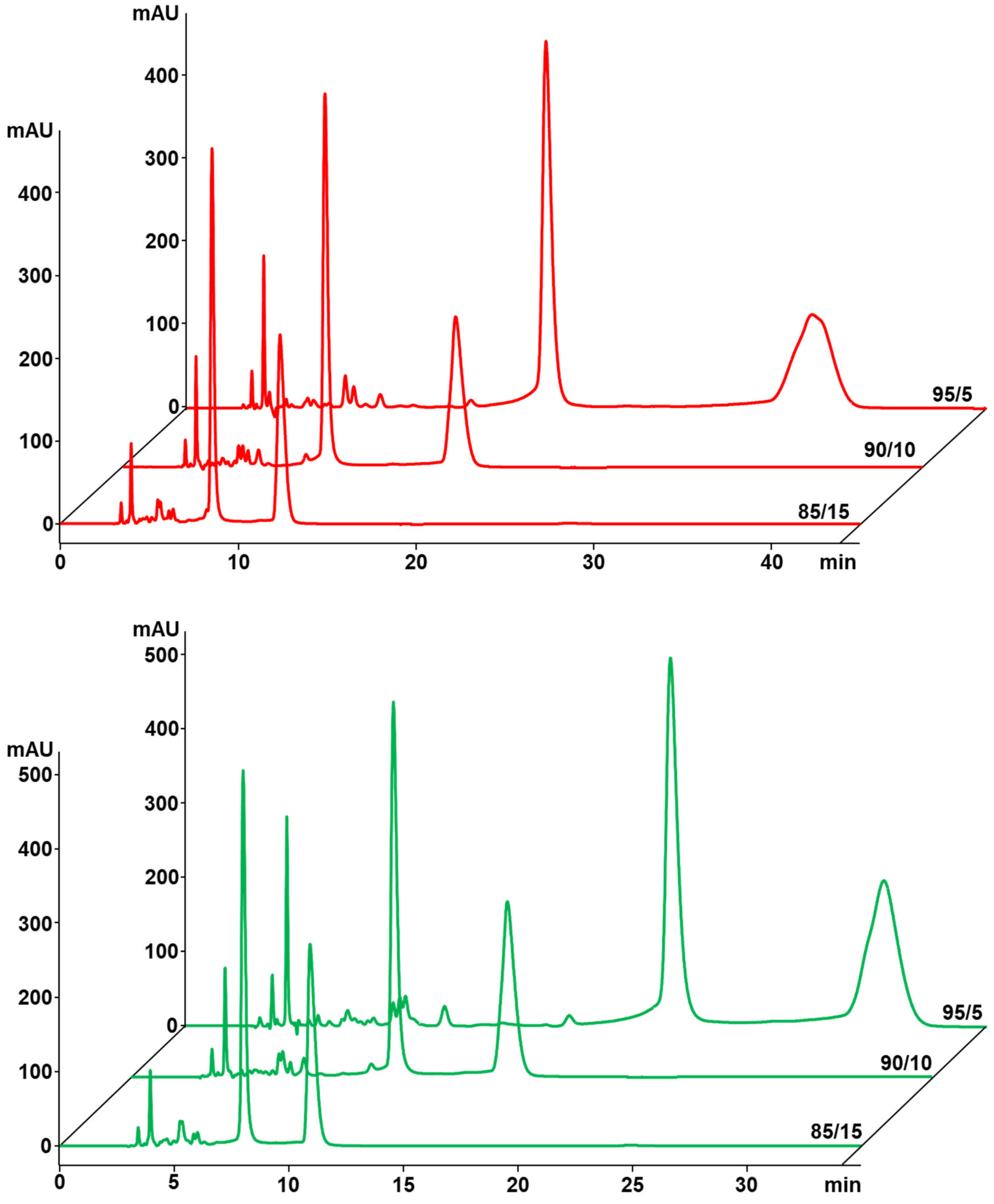

Figure S5: Chromatograms of IBMCat in heptane (red) and hexane (green) mobile phases measured on Amylose-SA.

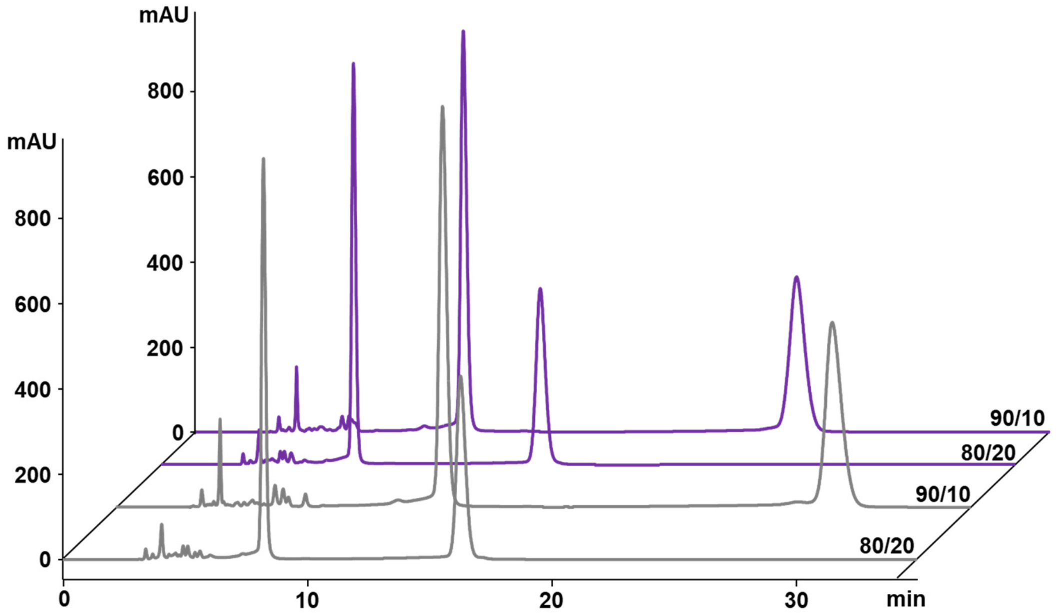

Figure S6: Chromatograms of IBMCat in heptane (purple) and hexane (grey) mobile phases with ethanol as polar modifier measured on column Amylose-SA.

Moreover, enantioseparation on experimental strong chiral cation-exchange columns developed in our laboratory was performed [50]. Chiral separation of compounds **4** and **6** was performed at temperature 25 °C with a flow of 1 mL·min^{-1}, an injection volume of 10 µl, and a detection wavelength of 254 nm. Columns: CSP I and CSP II (Fig. S7) and mobile phase acetonitrile/methanol/trifluoroacetic acid (TFA)/diethylamine (9/1, v/v + 50mM TFA + 25mM DEA). However, the separation was not successful (Fig. S8), which prompted us to use polysaccharide columns for preparative scale.

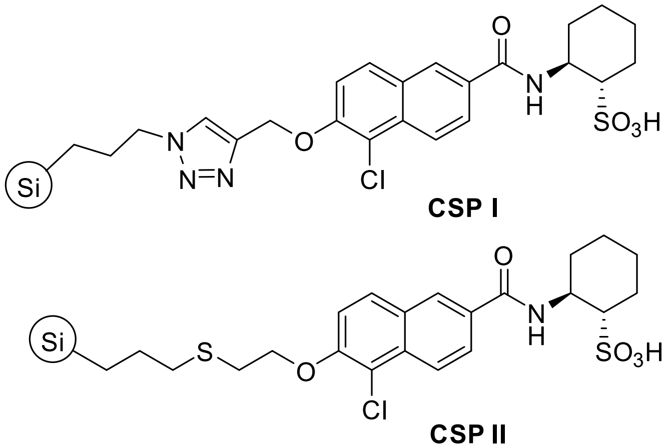

Figure S7: Chemical structure of chiral strong cation-exchange sorbents in CSP I and CSP II.

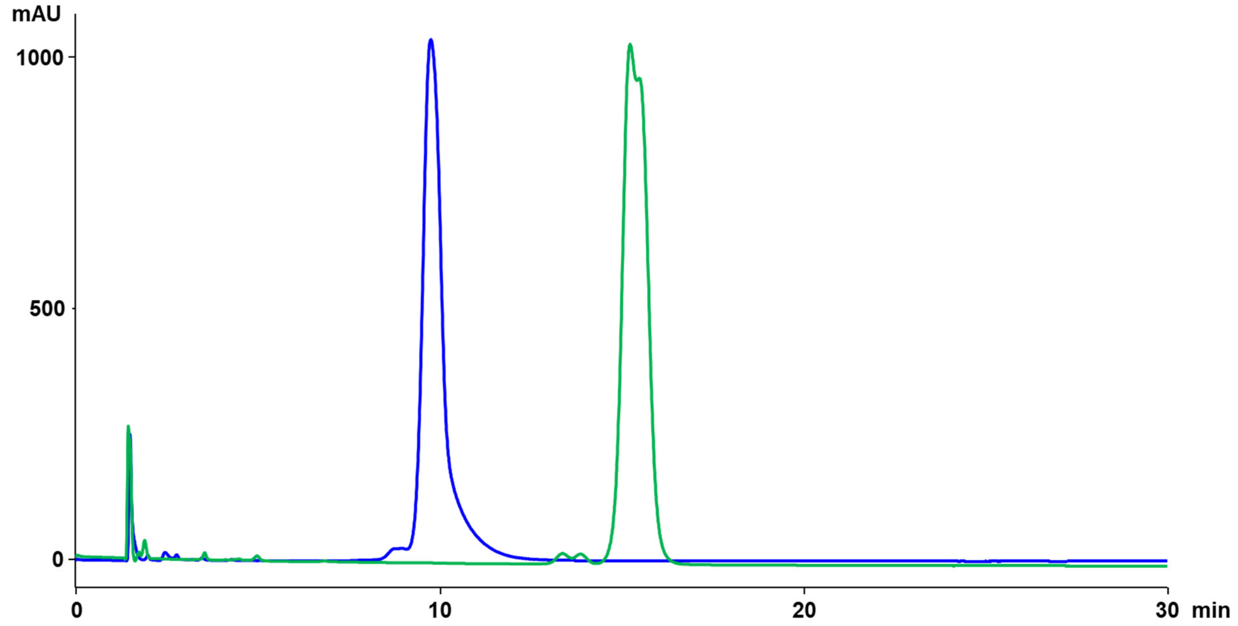

Figure S8: Chromatogram of IBMCat on cation-exchange columns CSP I (blue) and CSP II (green).

3. DFT calculations

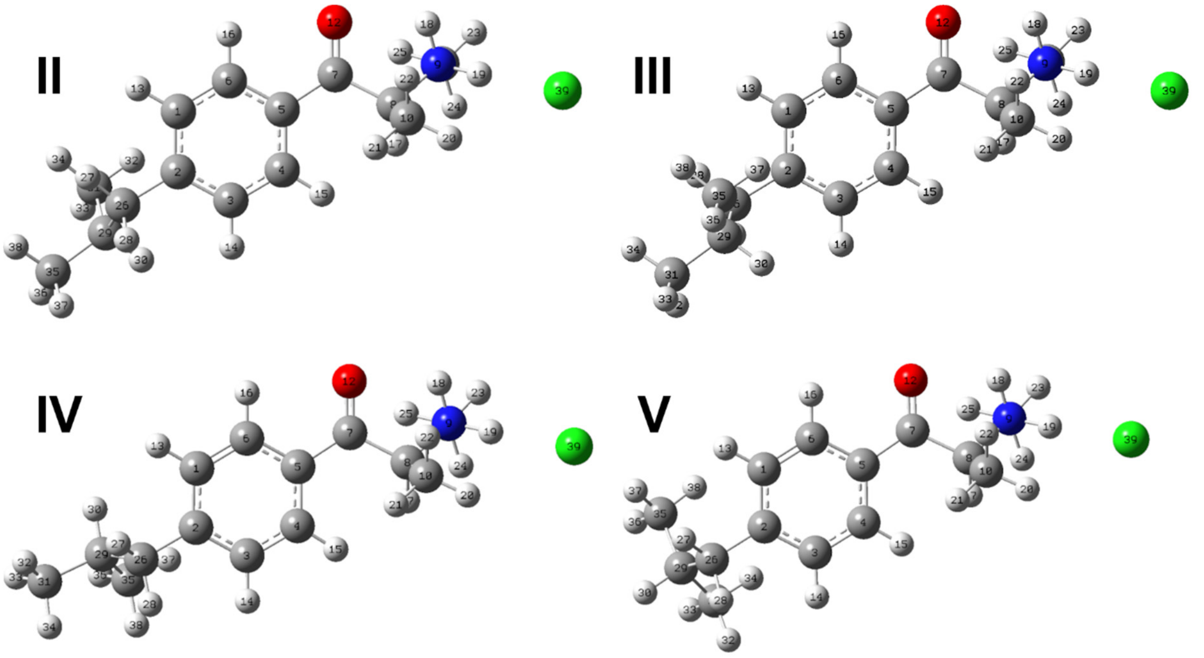

Figure S9: The structures of the stable conformers II, III, IV and V of 4-isobutylmethcathinone hydrochloride in MeOH simulated at B3LYP/6-311++G(d,p)/CPCM level.

4. Spectroscopic data

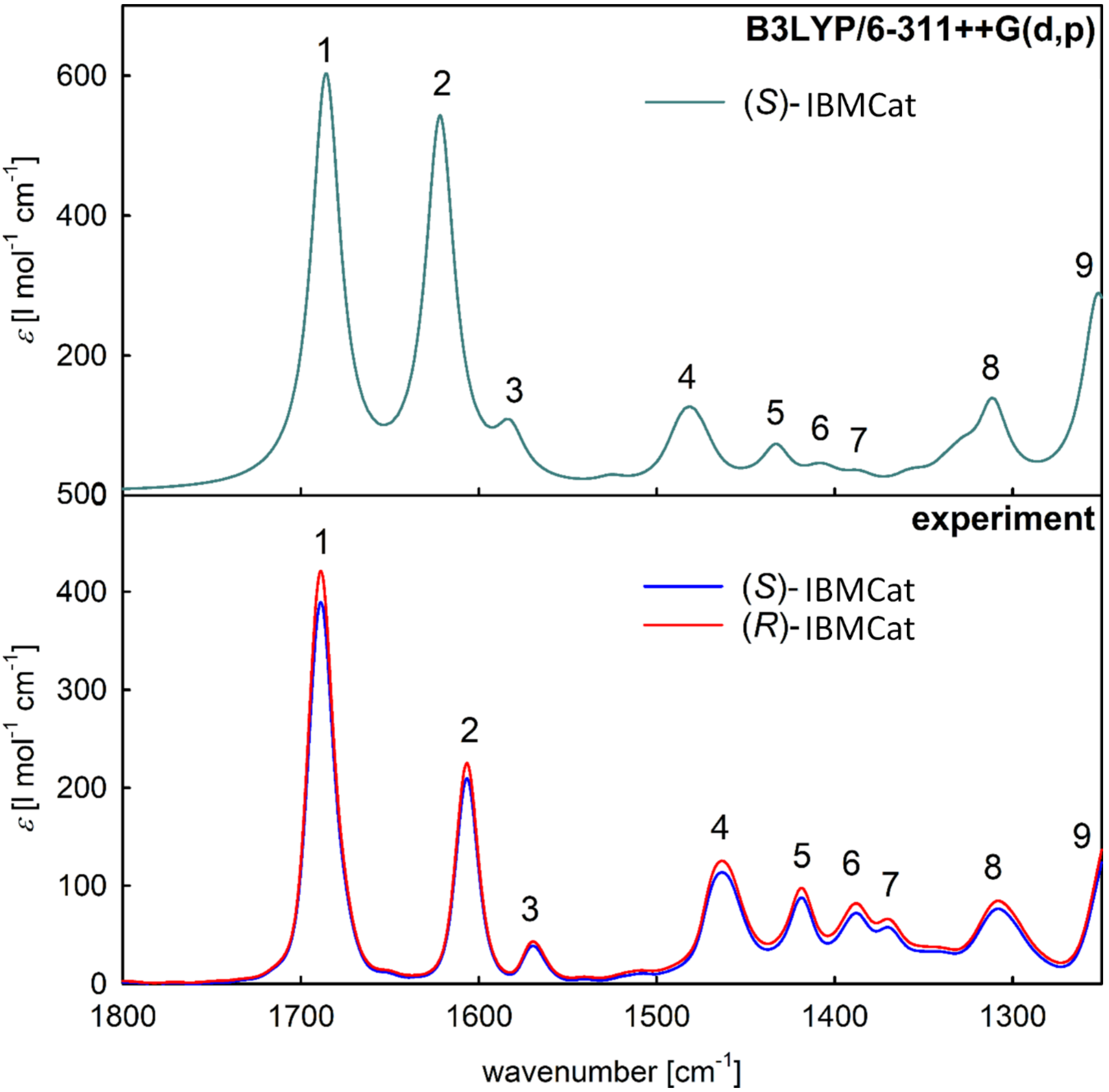

Figure S10: Comparison of the IR absorption spectra of enantiomers of IBMCat in MeOD-d_4: the spectrum simulated at the B3LYP/6-311++G(d,p) level (top) and experimental spectra (bottom).

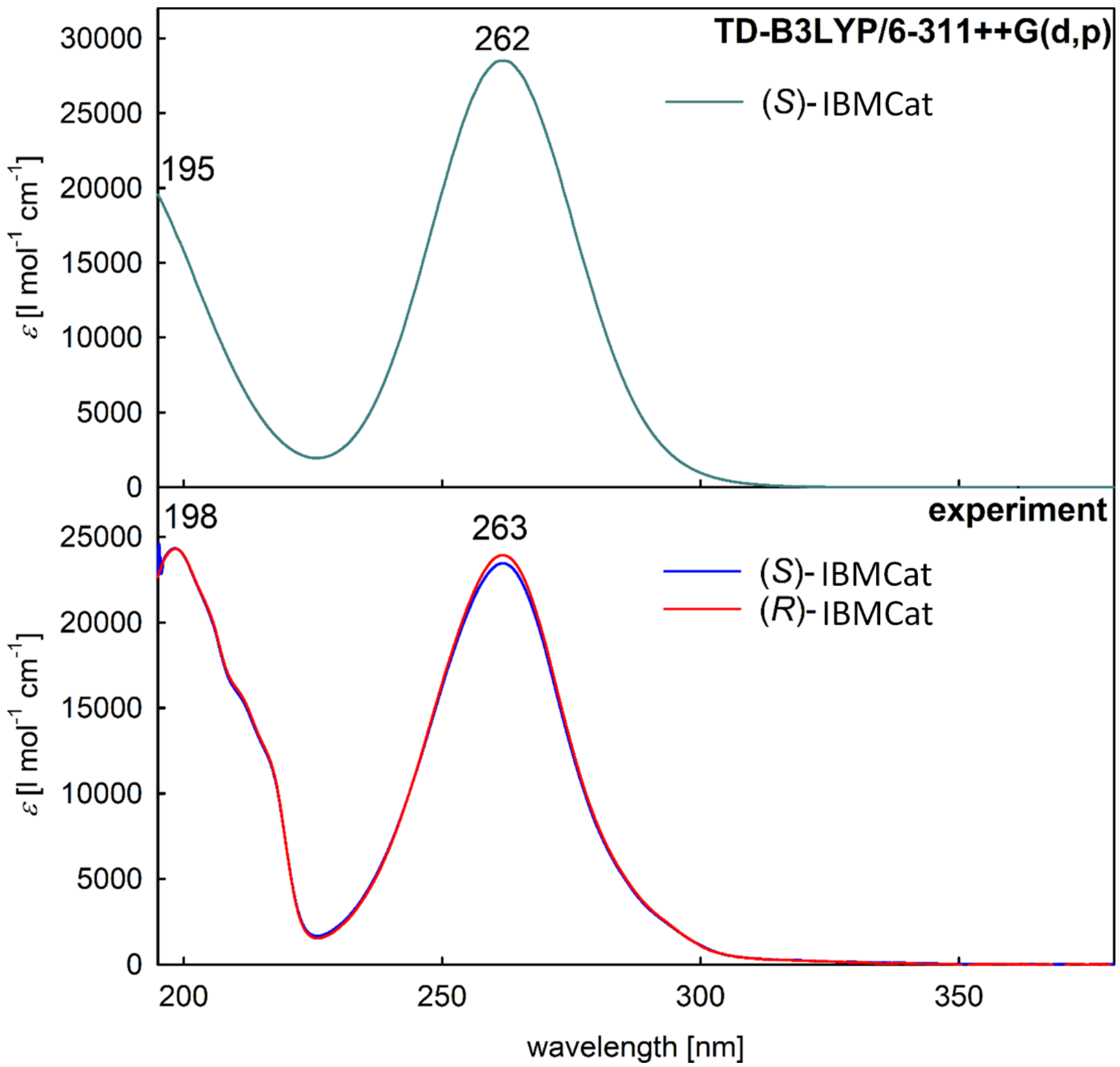

Figure S11: Comparison of the UV absorption spectra of enantiomers of IBMCat in MeOH: the simulated spectrum at the TD-B3LYP/6-311++G(d,p) level (top) and experimental spectra (bottom).

5. **Biological studies**

Table S3: Plasmid DNA and its source used in PRESTO-Tango β-arrestin recruitment assay

DRD1-Tango was a gift from Bryan Roth (Addgene plasmid #66268; http://n2t.net/addgene:66268; RRID: Addgene_66268)
DRD2-Tango was a gift from Bryan Roth (Addgene plasmid #66269; http://n2t.net/addgene:66269; RRID: Addgene_66269)
DRD3-Tango was a gift from Bryan Roth (Addgene plasmid #66270; http://n2t.net/addgene:66270; RRID: Addgene_66270)
DRD4-Tango was a gift from Bryan Roth (Addgene plasmid #66271; http://n2t.net/addgene:66271; RRID: Addgene_66271)
DRD5-Tango was a gift from Bryan Roth (Addgene plasmid #66272; http://n2t.net/addgene:66272; RRID: Addgene_66272)
ADRA2A-Tango was a gift from Bryan Roth (Addgene plasmid #66216; http://n2t.net/addgene:66216; RRID: Addgene_66216)
ADRA2B-Tango was a gift from Bryan Roth (Addgene plasmid #66217; http://n2t.net/addgene:66217; RRID: Addgene_66217)
ADRA2C-Tango was a gift from Bryan Roth (Addgene plasmid #66218; http://n2t.net/addgene:66218; RRID: Addgene_66218)
ADRB1-Tango was a gift from Bryan Roth (Addgene plasmid #66219; http://n2t.net/addgene:66219; RRID: Addgene_66219)
ADRB2-Tango was a gift from Bryan Roth (Addgene plasmid #66220; http://n2t.net/addgene:66220; RRID: Addgene_66220)
HTR2A-Tango was a gift from Bryan Roth (Addgene plasmid #66409; http://n2t.net/addgene:66409; RRID: Addgene_66409)
HTR2B-Tango was a gift from Bryan Roth (Addgene plasmid #66410; http://n2t.net/addgene:66410; RRID: Addgene_66410)
HTR2C-Tango was a gift from Bryan Roth (Addgene plasmid #66411; http://n2t.net/addgene:66411; RRID: Addgene_66411)

6. Receptor studies

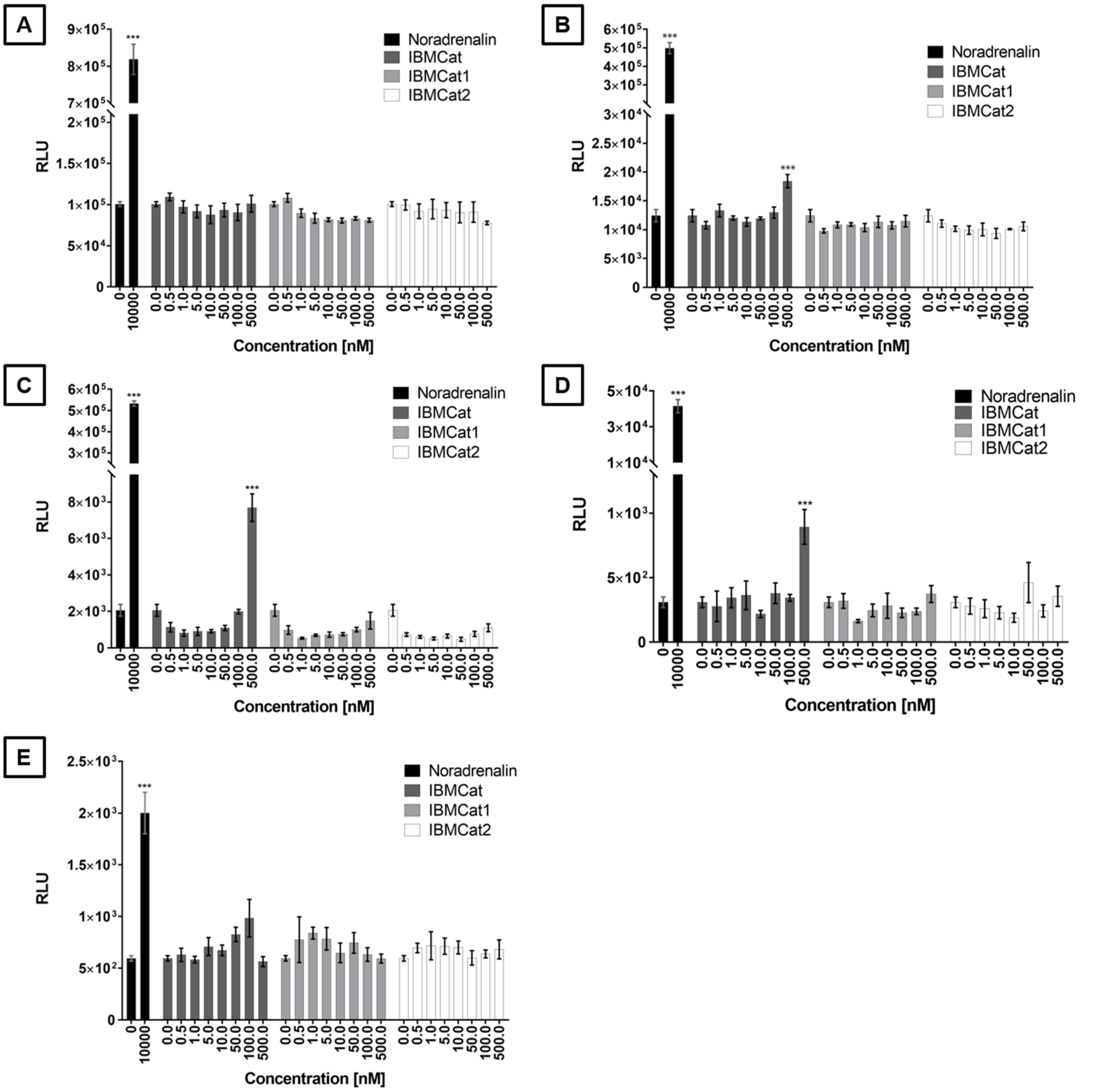

Figure S12: β-arrestin 2 recruitment PRESTO-Tango assay: activation of the adrenergic receptors. The plots represent the luminiscence level of oxyluciferin (relative light unit, RLU) arisen in HTLA cells transfected with pDNA encoding the adrenergic receptors and treated with 4-isobutylmethcathinone (IBMCat) and its enantiomers IBMCat1 (*S*-enantiomer) and IBMCat2 (*R*-enantiomer) at 0-500 nM concentration. As a control, a ligand of adrenergic receptors (ADRA), noradrenalin (10 µM concentration), was used. A) ADRA2A (alpha-2A adrenergic receptor), B) ADRA2B (alpha-2B adrenergic receptor), C) ADRA2C (alpha-2C adrenergic receptor), D) ADRB1 (beta-1 adrenergic receptor), and E) ADRB2 (beta-2 adrenergic receptor). The error bar represents standard deviation from four replicates. The data were processed and evaluated by GraphPad Prism Software using one-way ANOVA: * $p<0.05$; ** $p<0.01$; *** $p<0.001$.

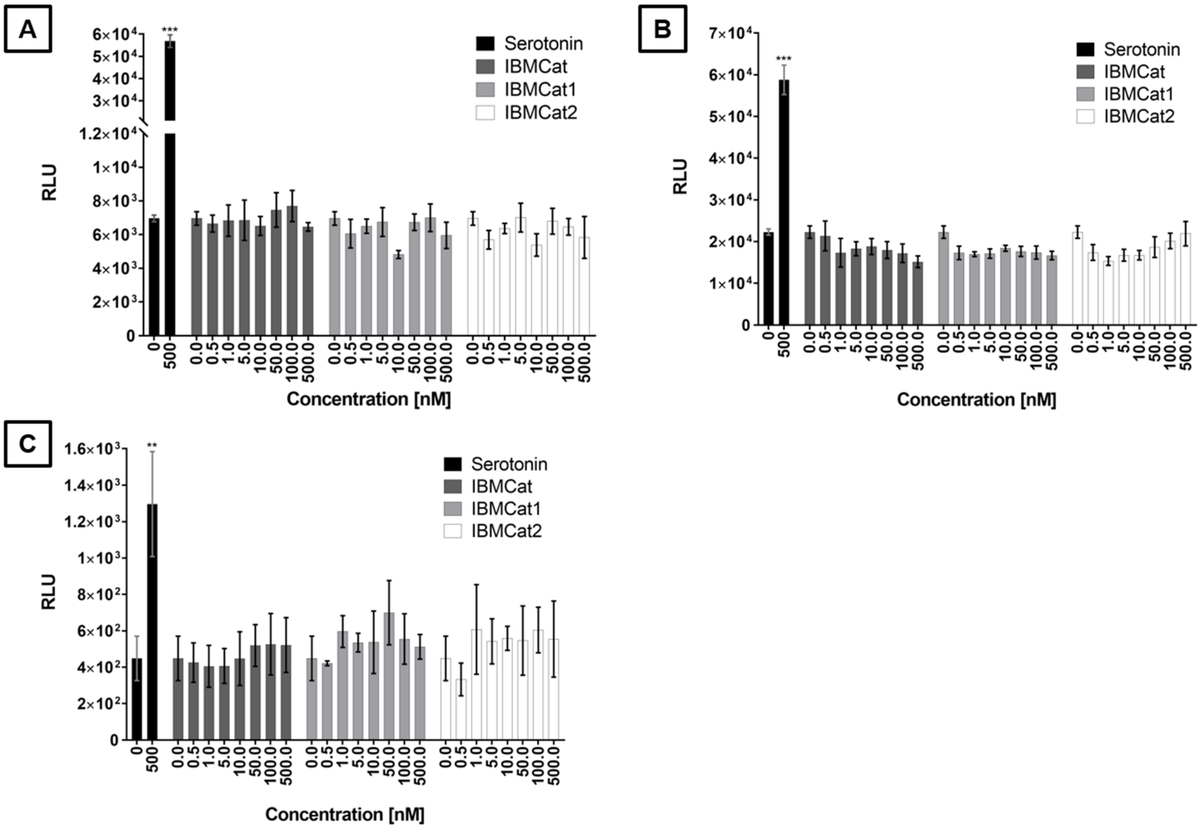

Figure S13: β-arrestin 2 recruitment PRESTO-Tango assay: activation of the serotonin receptors. The plots represent the luminiscence level of oxyluciferin (relative light unit, RLU) arisen in HTLA cells transfected with pDNA encoding the serotonin receptors and treated with 4-isobutylmethcathinone (IBMCat) and its enantiomers IBMCat1 (*S*-enantiomer) and IBMCat2 (*R*-enantiomer) at 0-500 nM concentration. As a control, a ligand of the serotonin receptors (HTR), serotonin (500 nM concentration), was used. A) HTR2A (5-hydroxytryptamine receptor 2A), B) HTR2B (5-hydroxytryptamine receptor 2B), and C) HTR2C (5-hydroxytryptamine receptor 2C). The error bar represents standard deviation from four replicates. The data were processed and evaluated by GraphPad Prism Software using one-way ANOVA: * $p<0.05$; ** $p<0.01$; *** $p<0.001$.

References

46. Sawada, K.; Okada, S.; Kuroda, A.; Watanabe, S.; Sawada, Y.; Tanaka, H. 4-(Benzoylindolizinyl)butyric Acids; Novel Nonsteroidal Inhibitors of Steroid 5α-Reductase. III. *Chem. Pharm. Bull.* **2001**, *49*, 799-813. https://doi.org/10.1248/cpb.49.799.

47. King, C.L.; Ostrum, K.G. Selective bromination with copper(II) bromide. *J. Am. Chem. Soc.* **1964**, *29*, 3459-3461. https://doi.org/10.1021/jo01035a003.

28. Spálovská, D.; Paškan, M.; Jurásek, B.; Kuchař, M.; Kohout, M.; Setnička, V. Structural spectroscopic study of enantiomerically pure synthetic cathinones and their major metabolites. *New J. Chem.* **2021**, *45*, 850–860. https://doi.org/10.1039/D0NJ05065B.

48. Malmedy, F.; Wirth, T. Stereoselective Ketone Rearrangements with Hypervalent Iodine Reagents. *Chem. Eur. J.* **2016**, *22*, 16072–16077. https://doi.org/10.1002/chem.201603022.

49. Sonawane, H. R.; Rellur, N.S.; Kulkarni, D.G.; Ayyangar, N.R. Photochemical Rearrangement of α-Chloro-Propiophenones to α-Arylpropanoic Acids: Studies on Chirality Transfer and Synthesis of (S)-(+)-Ibuprofen and (S)-(+)-Ketoprofen. *Tetrahedron* **1994**, *50*, 1243-1260. https://doi.org/10.1016/S0040-4020(01)80835-8.

50. Hercikova, J.; Spálovská, D.; Frühauf, P.; Izák, P.; Lindner, W.; Kohout, M. Design and synthesis of naphthalene-based strong cation exchangers and their application for chiral separation of basic drugs. J. Sep. Sci. 2021, 44, 3348-3356. https://doi.org/10.1002/jssc.202100127.

Appendix

^{1}H and ^{13}C spectra of compounds **2-6**.

1-(4-Isobutylphenyl)propan-1-one (2)

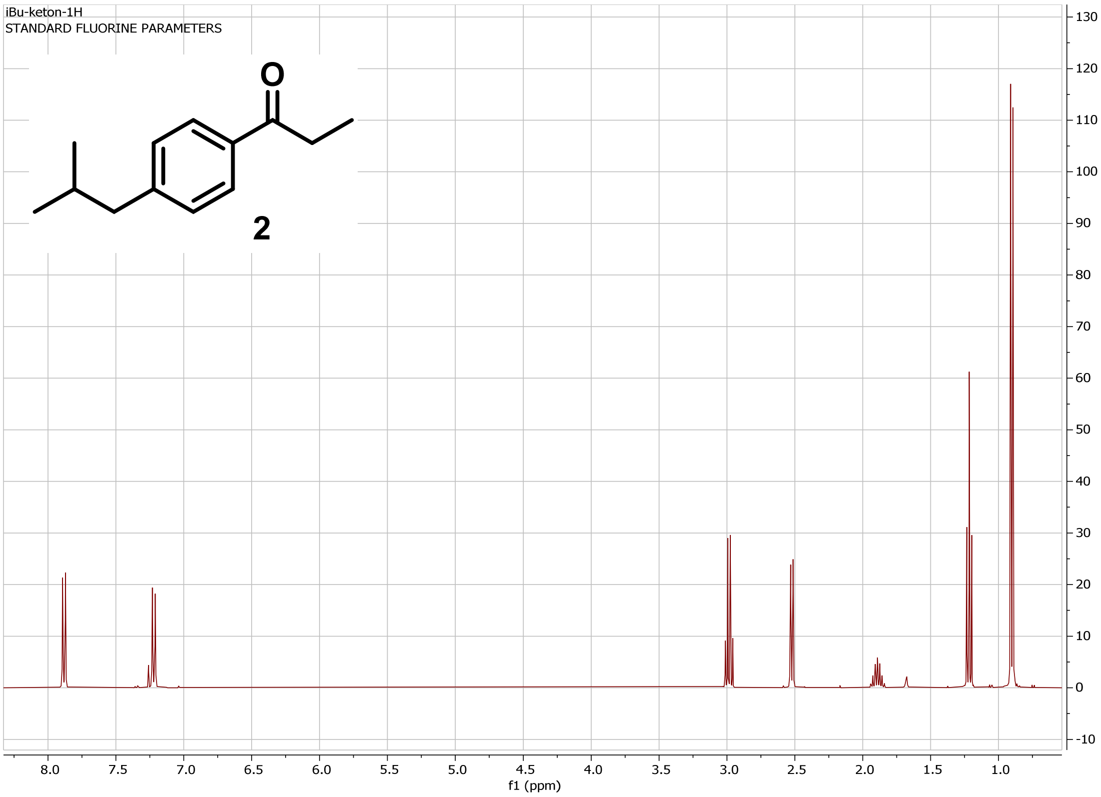

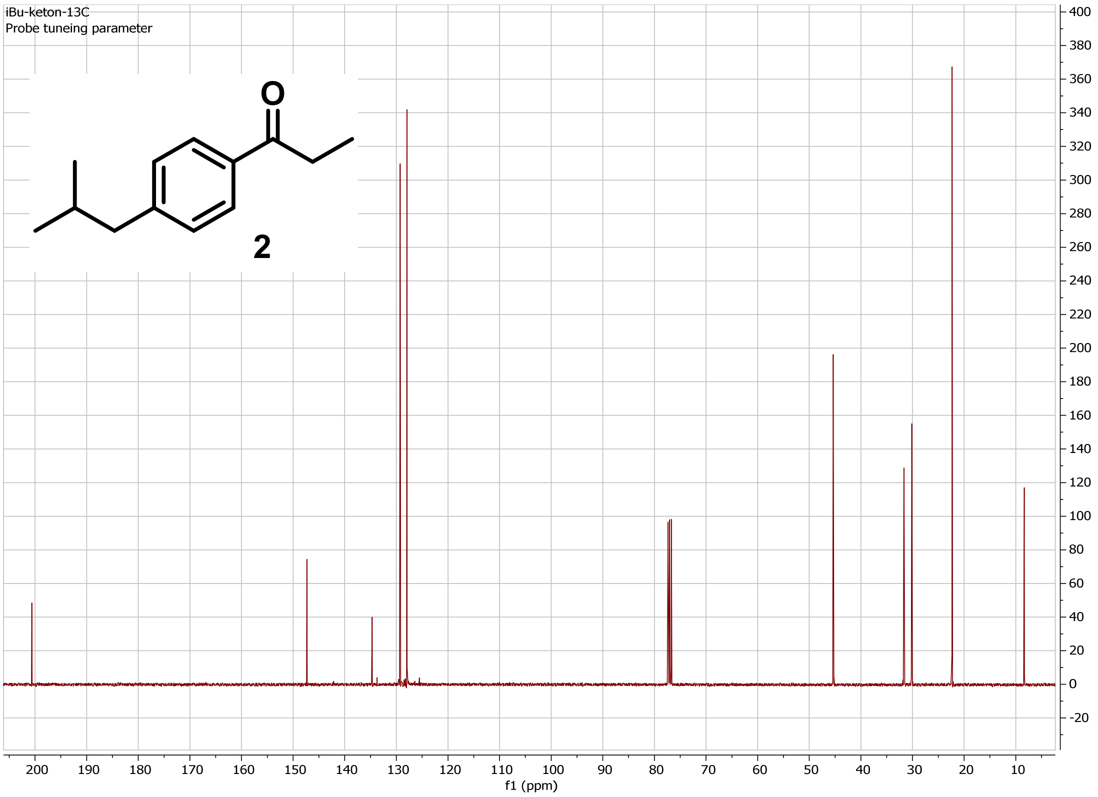

2-Bromo-1-(4-isobutylphenyl)propan-1-one (3)

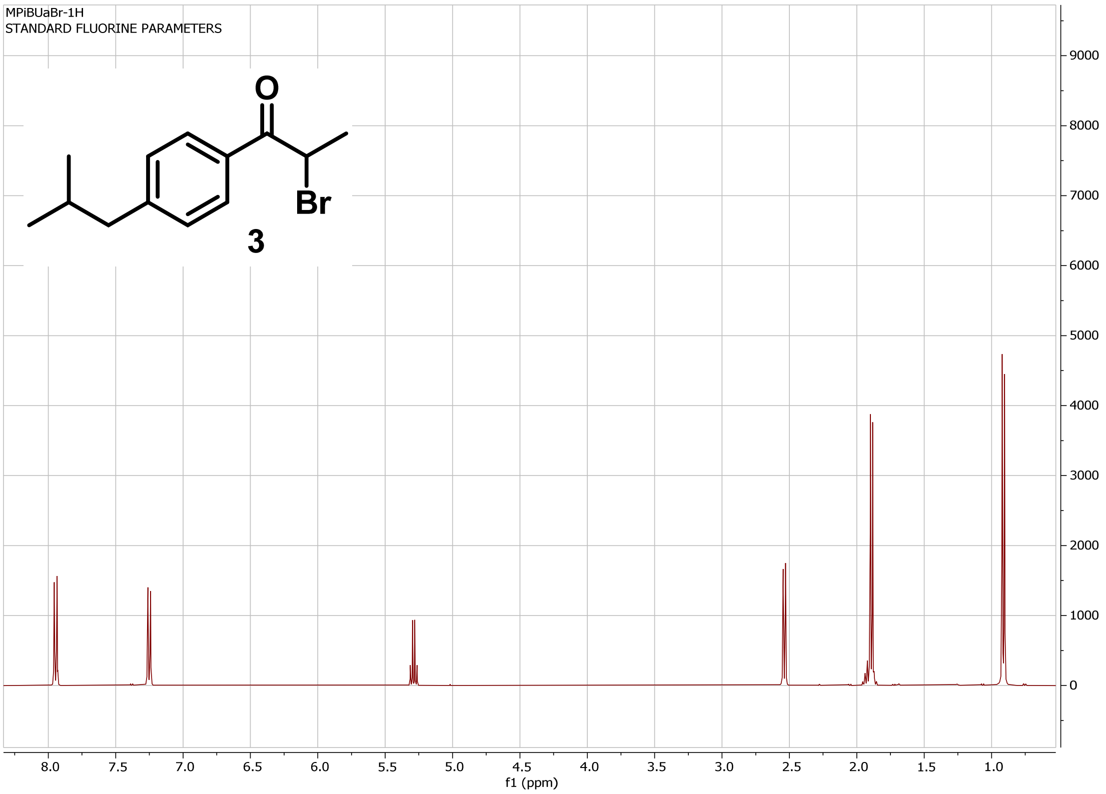

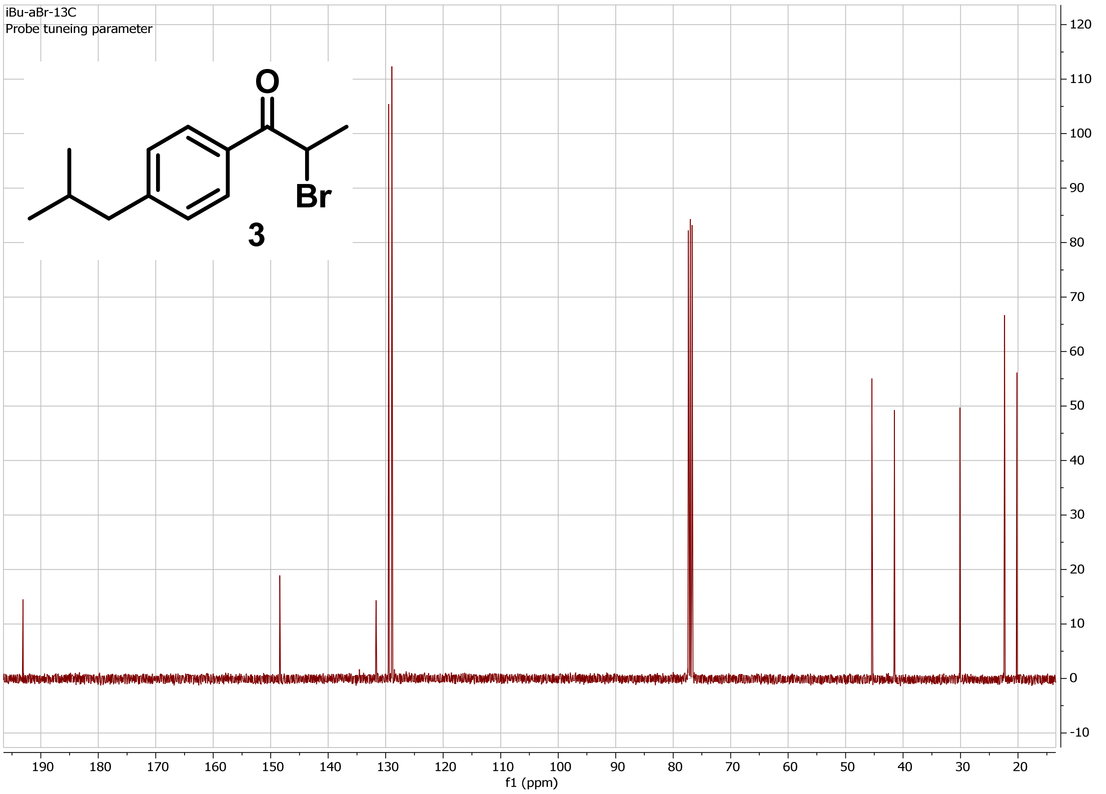

1-(4-Isobutylphenyl)-2-(methylamino)propan-1-one hydrochloride (4)

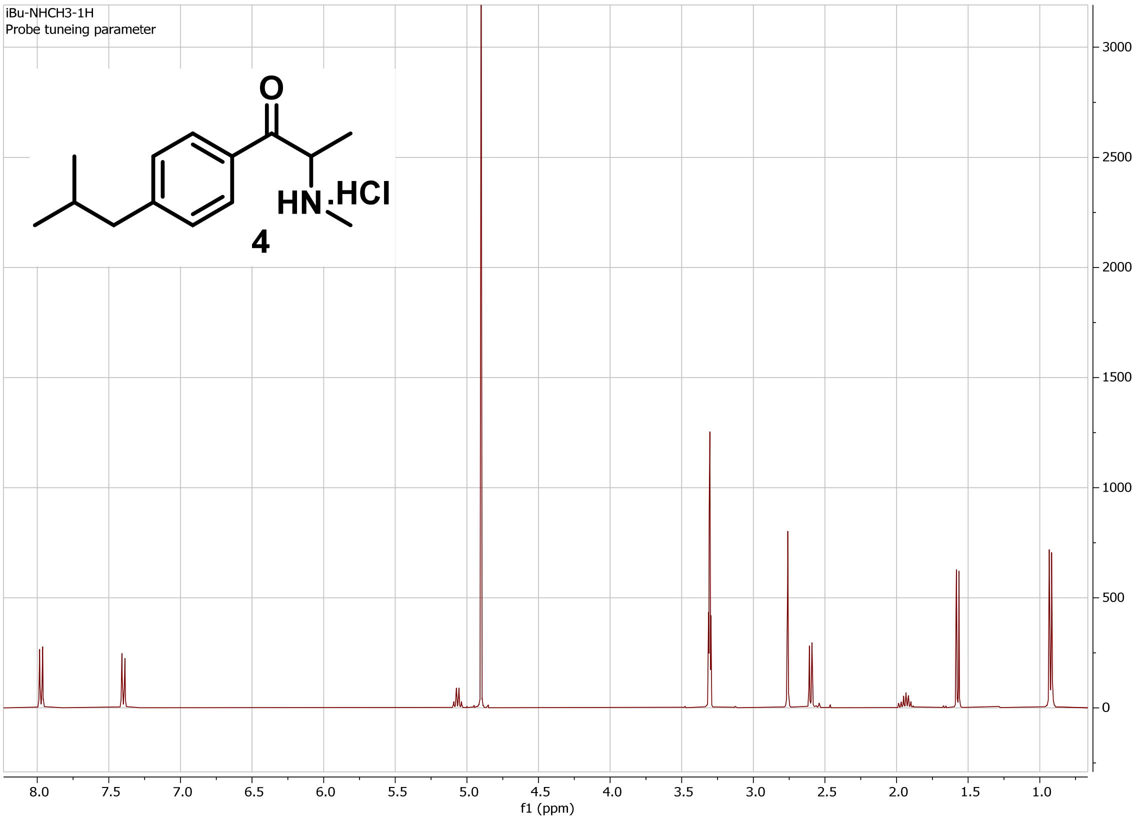

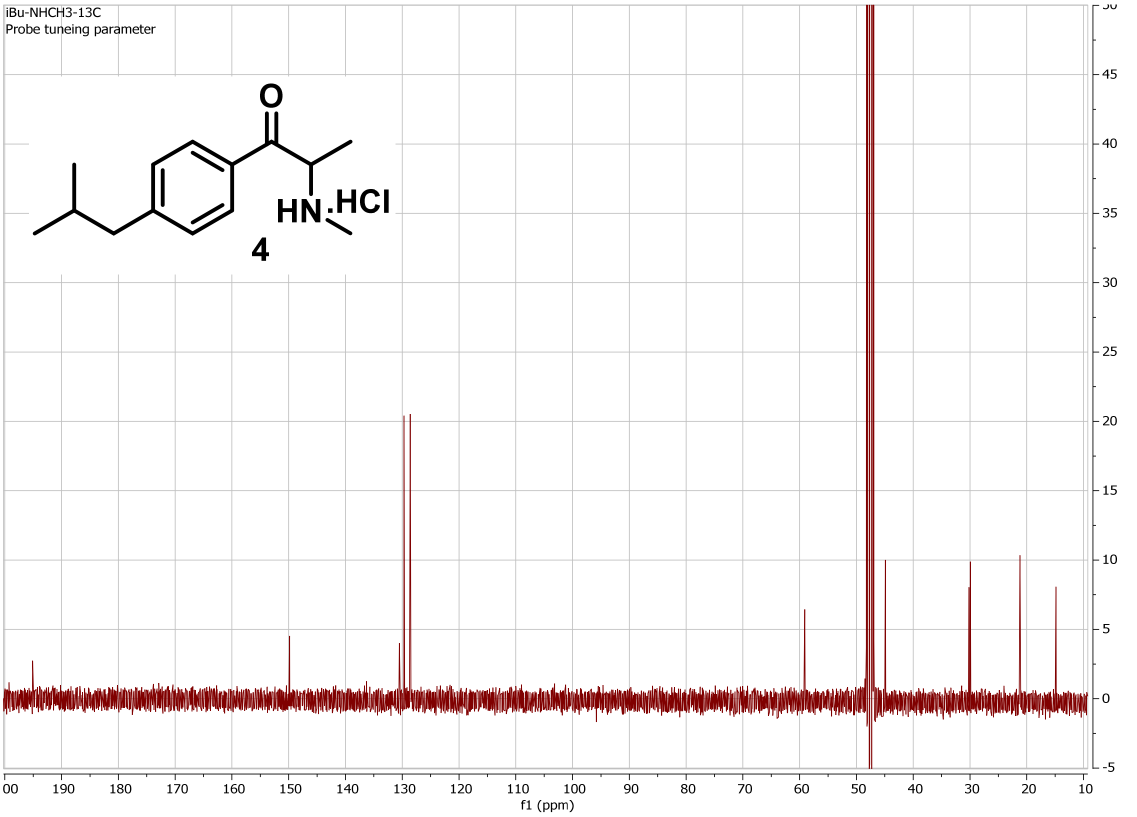

2-Azido-1-(4-isobutylphenyl)propan-1-one (5)

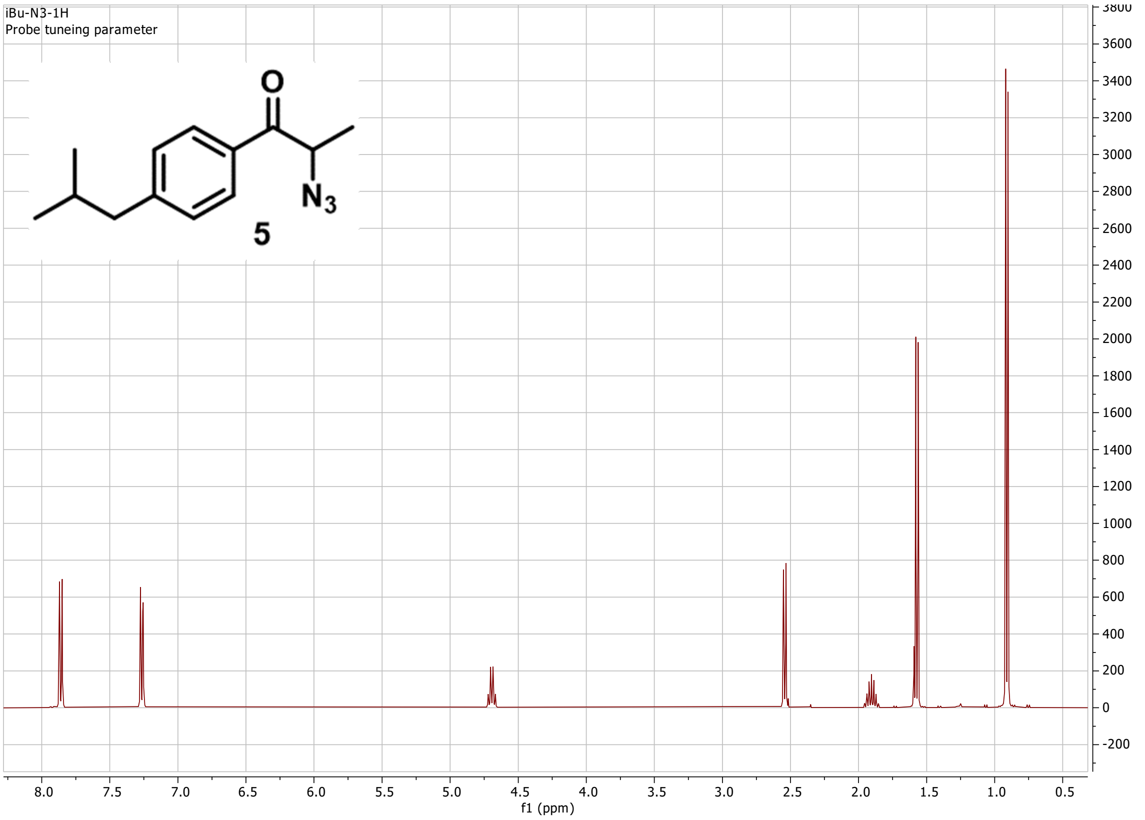

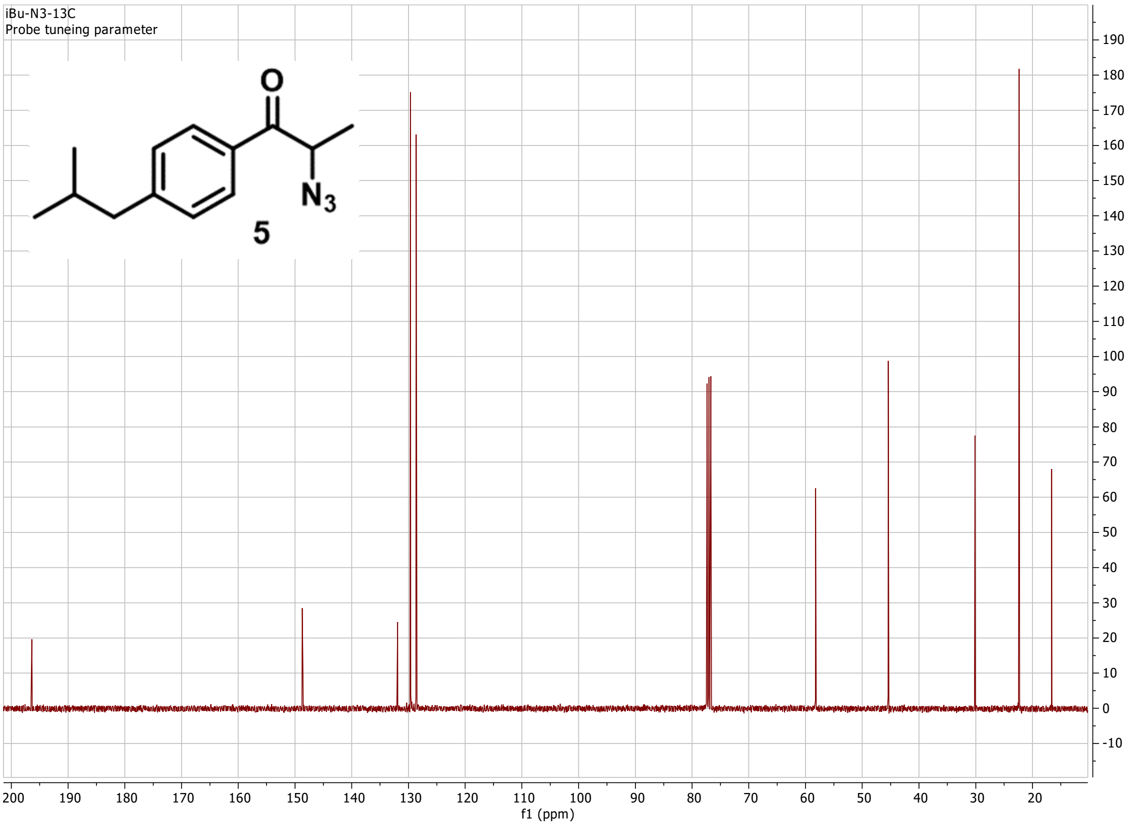

2-Amino-1-(4-isobutylphenyl)propan-1-one hydrochloride (6)

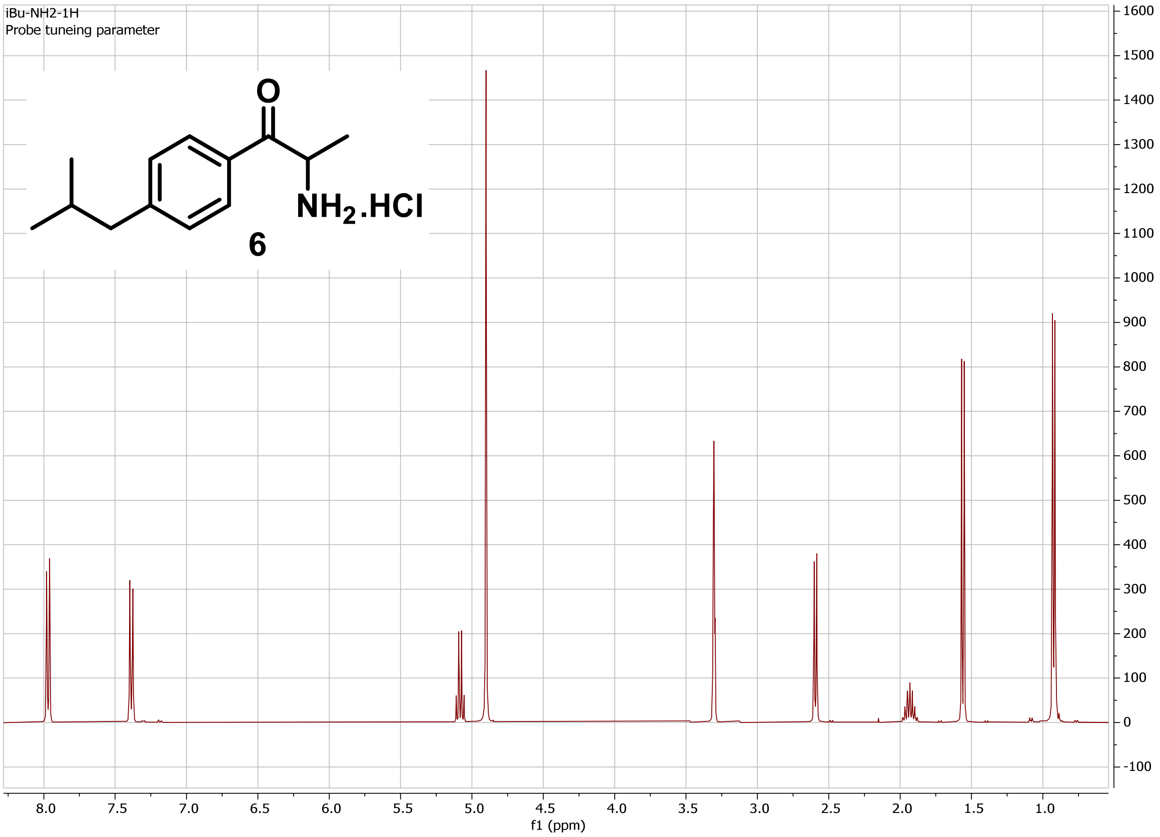

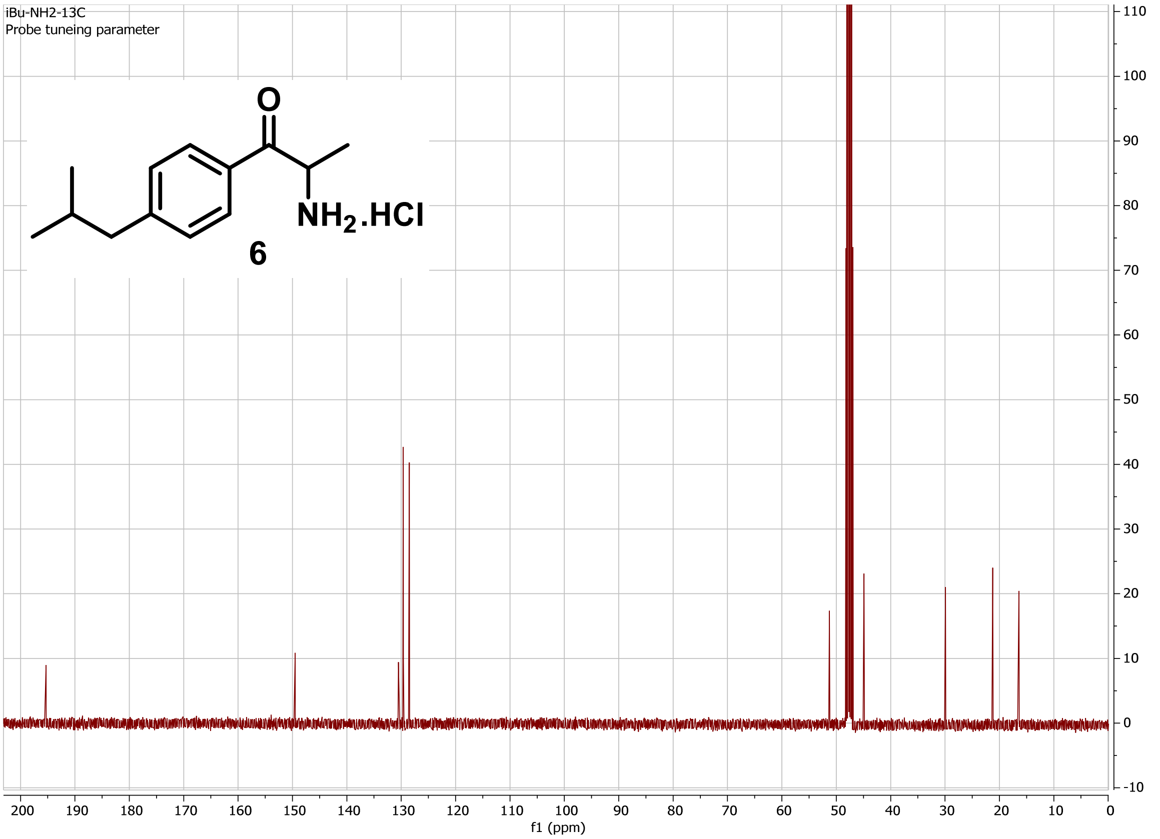

Supplement: Supplementary file 1 [file pharmaceuticals-15-01495-s001.zip › pharmaceuticals-2015339-supplementary.pdf]
